# The genome sequence of *Brucella pinnipedialis *B2/94 sheds light on the evolutionary history of the genus *Brucella*

**DOI:** 10.1186/1471-2148-11-200

**Published:** 2011-07-11

**Authors:** Stéphane Audic, Magali Lescot, Jean-Michel Claverie, Axel Cloeckaert, Michel S Zygmunt

**Affiliations:** 1 Laboratoire Information Génomique et Structurale, CNRS-UPR2589, Aix-Marseille University, Institut de Microbiologie de la Méditerranée (IM2, IFR-88), Parc Scientifique de Luminy-163 Avenue de Luminy-Case 934-FR-13288, Marseille cedex 09, France; 2 CNRS, UMR 7144, Equipe Evolution du Plancton et Paléo-Océans, Station Biologique de Roscoff, 29682 Roscoff, France; 3 UPMC Univ Paris 06, UMR 7144, Adaptation et Diversité en Milieu Marin, Station Biologique de Roscoff, 29682 Roscoff, France; 4 INRA, UR1282, Infectiologie Animale et Santé Publique, IASP, Nouzilly, F-37380, France

**Keywords:** Brucella, Bacterial Genome Evolution, Comparative Genomics

## Abstract

**Background:**

Since the discovery of the Malta fever agent, *Brucella melitensis*, in the 19th century, six terrestrial mammal-associated *Brucella *species were recognized over the next century. More recently the number of novel *Brucella *species has increased and among them, isolation of species *B. pinnipedialis *and *B. ceti *from marine mammals raised many questions about their origin as well as on the evolutionary history of the whole genus.

**Results:**

We report here on the first complete genome sequence of a *Brucella *strain isolated from marine mammals, *Brucella pinnipedialis *strain B2/94. A whole gene-based phylogenetic analysis shows that five main groups of host-associated *Brucella *species rapidly diverged from a likely free-living ancestor close to the recently isolated *B. microti*. However, this tree lacks the resolution required to resolve the order of divergence of those groups. Comparative analyses focusing on a) genome segments unshared between *B. microti *and *B. pinnipedialis*, b) gene deletion/fusion events and c) positions and numbers of *Brucella *specific IS*711 *elements in the available *Brucella *genomes provided enough information to propose a branching order for those five groups.

**Conclusions:**

In this study, it appears that the closest relatives of marine mammal *Brucella *sp. are *B. ovis *and *Brucella *sp. NVSL 07-0026 isolated from a baboon, followed by *B. melitensis *and *B. abortus *strains, and finally the group consisting of *B. suis *strains, including *B. canis *and the group consisting of the single *B. neotomae *species. We were not able, however, to resolve the order of divergence of the two latter groups.

## Background

Brucellae are Gram-negative, facultative, intracellular bacteria that can infect many species of animals and man. Six species were initially recognized within the genus *Brucella: B. abortus, B. melitensis, B. suis, B. ovis, B. canis*, and *B. neotomae *[1-3]. This classification is mainly based on differences in pathogenicity, host preference, and phenotypic characteristics. Four additional species have been included in the genus *Brucella *since 2007. These comprise the species *B. ceti *and *B. pinnipedialis *isolated from marine mammals, with cetaceans (dolphin, porpoise, and whale species) and pinnipeds (various seal species) as preferred host respectively [4,5]. *B. microti *described in 2008 was first isolated from the common vole and then from the red fox, and from soil [6-8]. The latest described species is *B. inopinata*, isolated from an infected human breast implant, and currently the most divergent *Brucella *species at the phenotypic and molecular level [9,10]. The animal or environmental reservoir of *B. inopinata *is not known. New *Brucella *species will likely be described in the future such as for isolates from baboons [11], from wild rodents in Australia [12] and for strain BO2 isolated from a patient with chronic destructive pneumonia [13]. Strain BO2 and strains from wild Australian rodents have been proposed as a novel lineage of the *B. inopinata *species [12,13].

Molecular and phenotypic typing of marine mammal *Brucella *strains led to their classification into two species, *B. ceti *and *B. pinnipedialis*, according to their preferred host, cetaceans and pinnipeds respectively [5]. However, several subgroups were identified within each species by molecular typing methods such as multilocus sequence analysis (MLSA), multilocus VNTR (Variable Number of Tandem Repeats) analysis (MLVA), or *omp2a *and *omp2b *porin genes [14-19]. Among them one subgroup within *B. ceti*, exclusively composed of strains isolated from various dolphin species, was proposed to constitute a separate species with the name *B. delphini *[3,14,18]. The isolates from cetaceans from the Pacific may also constitute a separate species [19]. Three human cases with naturally acquired infection by *Brucella *strains presumably from marine origin were reported, one case of spinal osteomyelitis from a patient in New Zealand [20] and two neurobrucellosis cases from Peruvian patients [21]. Interestingly, these human isolates exhibited the same genotype as strains from cetaceans from the Pacific [22].

Among their distinctive characteristics at the molecular and genomic level, marine mammal *Brucella *strains were shown to carry in their genomes a higher number of the insertion sequence element IS*711 *(or IS*6501*) [23,24] than terrestrial mammal *Brucella *species and biovars with the exception of *B. ovis *[15,17,25]. Consequently, infrequent restriction site-PCR (IRS-PCR) methods and more recently ligation-mediated PCR (LM-PCR) were applied, taking into account this higher number of IS*711 *elements, to study the genomic diversity of marine mammal strains [26-28]. These studies confirmed the classification into two marine mammal *Brucella *species, each divided in subgroups. In addition, six specific IS*711*-containing DNA fragments were detected allowing the molecular identification of *B. ceti *and its subgroup composed exclusively of dolphin isolates [17,26-28]. Besides these specific IS*711*-containing fragments another DNA fragment was detected that was exclusively found in *B. pinnipedialis *strains, with the exception of hooded seal isolates, consisting of a putative genomic island [26,27]. The size of this island was estimated at 62 kbp according to the physical maps made from the genomes of marine mammal strains by macrorestriction analyses [14].

The taxonomy of *Brucella *is still controversial, with an ongoing debate on whether they should be considered as distinct species or distinct strains of *B. melitensis*, considering the close proximity of their genomes [29]. We determined and analyzed the complete genome of *B. pinnipedialis *B2/94 to bring new insights into the origin of *Brucella *isolated from marine mammals as well as their time of divergence from *Brucella *isolated from terrestrial animals.

## Results and Discussion

The genome sequence of *B. pinnipedialis *B2/94 was determined (30× coverage) by shotgun sequencing with the GS-FLX technology and the remaining gaps filled using the standard Sanger technology. Like that of other *Brucella *strains, the genome is composed of two circular chromosomes, of 2,138,342 bp (base-pairs) and 1,260,926 bp in lengths, respectively. Bioinformatic annotation predicted the presence of 3,342 protein coding genes, 55 tRNAs and 9 ribosomal RNAs. The comparison with the known genomes of other *Brucella *species revealed the presence of 90 pseudogenes. The 23S rDNA sequence of *B. pinnipedialis *B2/94 was found similar to that of other *Brucella *species, in contrast with the anomalous and unexpected 23S ribosomal RNA sequence previously described for *B. microti *[30].

### Genome structure and whole gene set phylogeny

**Figure 1:**
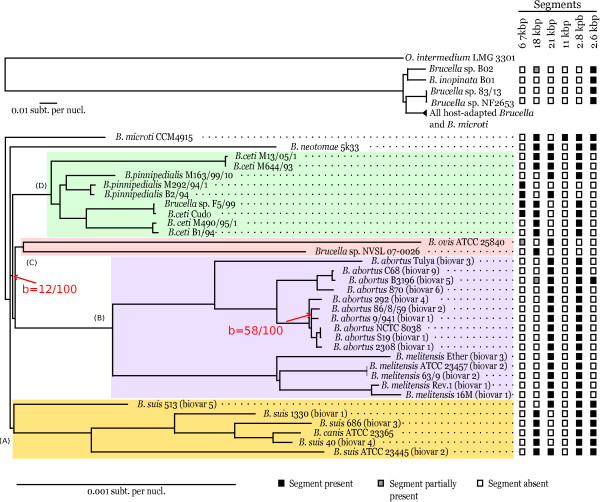
**Genus *Brucella *phylogenic tree based on a concatenated alignment of orthologous genes**. Phylogenetic representation of species in the genus *Brucella *based on a concatenated alignment of orthologous genes belonging to *Ochrobactrum intermedium *and 39 different *Brucella *strains or species whose genomes are nearly complete. At each leave of the tree is summarized the presence or absence pattern of the largest sequence fragments being unshared between *B. microti *CCM 4915 and *B. pinnipedialis *B2/94 (black square: presence, gray square: partial presence, white square: absence). Because of the very different scale of divergence of the different species in consideration in this work, the figure has been split in two parts. The upper part is a bird eye view showing the more distant *O. intermedium, Brucella *sp. B02*, B. inopinata, Brucella *sp. NF2653 and *Brucella *sp. 83/13 while the lower part displays the host-adapted *Brucella *species and *B. microti*. All nodes have bootstrap values above 99 (on 100 replicates) except when noted in red.

The phylogenetic placement of *B. pinnipedialis *B2/94 was assessed with the help of a whole gene tree including all orthologous genes from *Brucella *strains available in the complete genome division of GenBank, completed by sequence data from selected *Brucella *strains of particular phylogenetic relevance, either deposited in the whole genome shotgun (WGS) division of GenBank, or available from other sources (see Methods). This includes sequences from *B. neotomae*, 10 sequences from marine mammal *Brucella *strains, *Brucella *sp. B02, *B. inopinata *BO1, *Brucella *sp. 83/13 or *Brucella *sp. NF2653. The resulting tree (Figure 1) readily shows that the *Brucella *species with the highest sequence similarity with *B. pinnipedialis *B2/94 and whose complete genome is available is *B. microti *CCM 4915. This is a consequence of the relatively slow evolution rate of those two species rather than reflecting a particular phylogenetic affinity The central position occupied by *B. microti*, just at the root of host-associated *Brucella *species, is also confirmed. This tree distinguishes five major groups, the *B. suis *strains (shaded rectangle A, Figure 1), the different *B. melitensis *and *B. abortus *strains, also well separated from each other (rectangle B), another group with *B. ovis *and *Brucella *sp. NVSL 07-0026 (rectangle C), and marine mammal *Brucella *strains (rectangle D). *B. neotomae *remains isolated in this tree and constitutes the fifth group. However, the resolution of this tree is insufficient to infer the relative order of divergence of those different groups. Moreover, while bootstrap values in this tree are in general very strong (99 or 100%), the branching (D,(C, B)) proposed in the maximum likelihood tree is generally not supported, with a bootstrap value of 12%, prompting for a finer analysis of the events shaping the *Brucella *evolutionary tree.

The genome sequences of *B. microti *CCM 4915 and *B. pinnipedialis *B2/94 were found to be remarkably conserved even at the nucleotide level, allowing to generate a complete alignment of the chromosomes of the two species from which a list of all indels (insertions and deletions) (available as Additional file 1, Table S1 and Additional file 2, Table S2) was easily obtained. In spite of the overall similarity of these two genomes, this alignment revealed major changes in genome structure, as large segments unshared between the two species. The alignment from the largest chromosome exhibited 238 gapped positions with the largest insert in *B. microti *being 2,653 bp long, and the largest in *B. pinnipedialis *being 21,713 bp long. The alignment from the small chromosome exhibits 151 gapped positions, the largest insert being 18,341 bp long in *B. microti *and 67,389 bp long in *B. pinnipedialis*. The total number of indels (389) found in comparing these two species is thus smaller than the 405 indels found in comparing *B. microti *CCM 4915 and *B. suis *1330 [30]. The complete alignment contains a total of 3,290,621 aligned positions (2,104,923 and 1,185,698 per chromosome), with 0.10% of nucleotide changes in aligned regions (2,195 and 1,200 nucleotide changes, respectively). This fraction of nucleotide substitution is also smaller than what was observed between *B. microti *CCM 4915 and *B. suis *1330, where a 0.16% divergence was reported [30].

### Detailed analysis of the largest unshared sequence regions and the branching order of host associated *Brucella *species

The evolutionary history of the largest segments unshared between *B. microti *CCM 4915 and *B. pinnipedialis *B2/94 was analyzed by examining the structure of the orthologous loci in the other *Brucella *species for which sequence data was available (see Methods). On the large chromosome, the 2 largest indels are 21 kbp, and 2.6 kbp in length. On the small chromosome, the largest indels are 67 kbp, 18 kpb, 11 kbp and 2.8 kbp in length. All other indels are at most the size of an IS*711 *insertion sequence (843 bp). The presence/absence of the above genomic inserts was assessed in other *Brucella *strains (see Methods). For each segment, we recorded the number of nucleotides with homologues in the other *Brucella *genomes (Additional file 3, Table S3). The presence/absence of those segments is reported at the leaves of the tree (Figure 1) as filled squares. The evolutionary history of those unshared genome segments, treated as discrete characters, was reconstructed by parsimony analysis using the Mesquite software (see Methods), and represented as a cladogram in Additional file 4, Figure S1. The proposed evolutionary scenario corresponding to those unshared segments is discussed below, the largest ones first.

**Figure 2:**
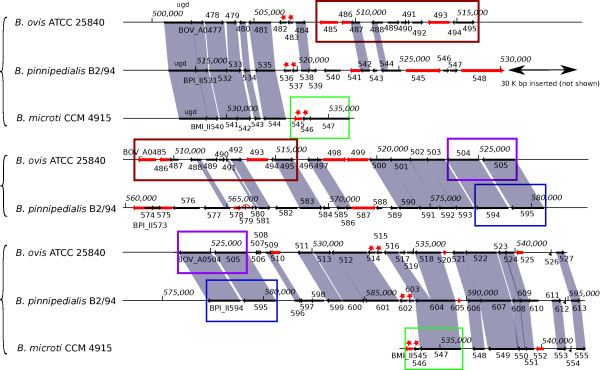
***B. pinnipedialis *B2/94 specific 67 kbp genome fragment and corresponding locus in *B. ovis *and *B. microti***. Schematic representation of the genome fragments surrounding the position of the 67 kbp insert in the genome of *B. pinnipedialis *B2/94, reduced to a 30 kbp fragment in *B. ovis *ATCC 25840 and absent from *B. microti *CCM 4915. Black arrows represent protein coding genes and red arrows pseudogenes. Orthologous protein coding genes present in several genomes are shown linked by grey parallelograms. Coordinates along the different genomes are also indicated. Locus names are abbreviated to their suffix, except for the first locus of a line. The red stars indicate the IS*711 *insertion element, present in a single copy in *B. microti *and in two copies, at both sides of the insert in *B. pinnipedialis *and *B. ovis*. Colored rectangles indicate groups of genes that are repeated in the representation to increase legibility of the drawing.

The locus of BMI_II545-6 in *B. microti *CCM 4915 is occupied in the *B. pinnipedialis *B2/94 small chromosome by a 67,389 bp insert (position: 520,136 to 587,524) which is present in a number of *Brucella *strains from marine mammals and partially in *B. ovis *ATCC 25840. The gene arrangements of this genomic region in *B. pinnipedialis, B. microti *and *B. ovis *are depicted in Figure 2. This region is flanked on both sides by an IS*711 *element (respectively BPI_II536-7 and BPI_II602-3), at a location where a sole copy of the IS*711 *element is found in *B. microti *CCM 4915 (BMI_II545-6), as in all *Brucella *strains lacking this insert. This region is partially found in *B. ovis *ATCC 25840, except in the interval between BPI_II545 and BPI_II576 deleted from *B. ovis*. The deletion in *B. ovis *ATCC 25840 occurred inside the gene BOV_A0488, whose 85 first amino acids are similar to BPI_II577 and whose last 100 amino acids are similar to BPI_II544. This interval exhibits a few protein coding genes or their remnants, with similarities mainly found in distant alphaproteobacteria i.e. excluding *Brucella *and *Ochrobactrum*. In particular, two pseudogenes, BPI_II545 and BPI_II548, separated by one IS*711 *element, appear to be the remnants of two consecutive *Paracoccus denitrificans *genes coding for 5-oxoprolinase (Pden_4408-9) and are preceded by an AraC-family transcriptional regulator (BPI_II544) with similarity into the same organism (Pden_4410). The same three genes are also found grouped together but in different orders in the genomes of several betaproteobacteria e.g *Bordetella avium *(BAV3293-5) or *Verminephrobacter eiseniae *(Veis_1518-20). Two consecutive genes, *etfA *(BPI_II555) and *etfB *(BPI_II556) are also present on the large chromosome with 100% nucleotide identity. We also observed 4 components of a spermidine/putrescine ABC-transporter (BPI_II564-7) found colinearly in the genomes of e.g. *Roseobacter denitrificans *(RD1_3862-5) or *Silicibacter pomeroyi *(SPOA381-4). We found similarities to two consecutive genes of *Parvibaculum lavamentivorans *(Plav_0386-7), similarities to Plav_0387 being noticed on both sides of the BPI_II574-5 IS*Bm1 *transposase gene. Other genes like the aldehyde dehydrogenase BPI_II571 have best matches in *Brucella *and *Ochrobactrum*, but with 73% similarity only at the amino acid level. Several transposase genes are also present. This large 67 kbp fragment probably found its origin in the common ancestor of the marine mammal *Brucella *species and *B. ovis*, as attested by its partial presence in *B. ovis*. The structure of this segment suggests a grouping of *B. ovis *with marine mammal *Brucella *sp. (Additional file 4, Figure S1-a). It may have been lost thereafter several times, in *Brucella *sp. strain NVSL 07-006 isolated from a baboon and along the *B. ceti *and *B. pinnipedialis *lineages, putatively by recombination of the two IS*711 *copies.

A 21,713 bp fragment on the large chromosome (position: 259,190 to 280,902 in *B. pinnipedialis *B2/94) is not found in *B. microti *CCM 4915. This fragment is present in all *B. melitensis *and *B. abortus *strains and in *B. neotomae*, but its occurrence is quite variable among *B. ceti *and *B. pinnipedialis *strains and even among *B. suis *strains, where it is observed in the earliest diverging *B. suis *513 and *B. suis *ATCC 23445, but absent otherwise. This fragment starts with a phage integrase gene (BPI_I248) and ends with a tRNA (BPI_I278) which was the likely insertion site. An IS*711 *element is inserted within a gene (BPI_II256) that remained intact in *B. melitensis *16 M (BMEI1694) and *B. ovis *ATCC 25840 (BOV_0245). This fragment encodes a flagellar protein FlgJ (BPI_I260). The other putative genes in this region have no convincing similarities to annotated proteins. This 21 kbp fragment (Additional file 4, Figure S1-b) probably entered the *Brucella *genomes after *B. microti *divergence, and disappeared separately on several branches. It confirms the grouping of *B. ceti *Cudo, *B. ceti *B1/94, *B. ceti *M490/95/1 and *Brucella *sp. F5/99. Its presence in the genome of *B. neotomae *5K33 and absence in *B. suis *strains except for *B. suis *ATCC 23445 and *B. suis *513 suggest its insertion prior to the divergence of the *B. suis *and *B. neotomae *lineage, and a subsequent loss in the *B. suis *lineage.

A 18,341 bp region on the small chromosome (between BMI_II357 and BMI_II381) is absent from the *B. pinnipedialis *B2/94 genome (position: 344,671-363,011 in *B. microti *CCM 4915). This region is present in *B. microti, B. suis, B. canis, B. ceti, Brucella *sp. F5/99, *B. neotomae *and absent in *B. abortus, B. melitensis, B. ovis *and *B. pinnipedialis*. Additionally, a partial match was found in the genome of *Brucella *sp. B02. In this region, the presence of genes encoding TraI-J proteins involved in bacterial conjugation can be noted. Like the above 67 kbp fragment, this 18 kbp fragment (Additional file 4, Figure S1-c) supports a divergence of the *B. melitensis/B. abortus *clade prior to the separation of *B. ovis, Brucella *sp. strain NVSL 07-006 and marine mammal *Brucella *strains. It might have appeared before *B. microti *divergence, and disappeared several times, in particular from the branch leading to the *B. abortus*/*B. melitensis *clade and from the branch leading to the *B. pinnipedialis *clade.

The 11 kbp region on the small chromosome is a phage related region discussed in [30] and unique to *B. microti *(position: 1,038,883-1,050,624 in *B. microti *CCM 4915). It will not be discussed further (Additional file 4, Figure S1-d).

Still on the small chromosome, a 2,881 bp region (position: 1,082,391-1,085,271 in *B. microti *CCM 4915) encodes genes BMI_II1086-8. This fragment (Additional file 4, Figure S1-e) appeared before *B. microti *divergence, and is absent from the branch leading to *B. ovis *and *Brucella *sp. strain NVSL 07-0026, but also from the branch leading to *B. pinnipedialis *M292/94/1 and *B. pinnipedialis *B2/94, separating those two species from *B. pinnipedialis *M163/99/10. Closer examination reveals that this 2.8 kbp fragment is absent in *B. ovis *because it belongs to a much larger 44.5 kbp region deleted from *B. ovis *[31] and also partially deleted from *Brucella *sp. NVSL 07-0026, where an approximately 30 kbp long region is missing (pos: 1,117,180 to 1143394 in *B. pinnipedialis *B2/94 small chromosome). This finding supports both grouping of the *B. pinnipedialis *M292/94/1 and B2/94 strains, and that of *B. ovis *with *Brucella *sp. strain NVSL 07-0026.

Between BMI_I949 and BMI_I953, there is a 2,653 bp region deleted from the *B. pinnipedialis *B2/94 large chromosome (position: 928,716-931,368 in *B. microti *CCM 4915) but also from *B. ovis *ATCC 25840, *Brucella *sp. NVSL 07-0026 and all *B. melitensis *and *B. abortus *strains. Interestingly, this deletion occurred inside a gene (encoding an ABC transporter), thus showing that it is a deletion and not an acquisition event. This region (Additional file 4, Figure S1-f) is particularly informative because it clearly separates *Brucella *strains into two groups. This fragment was present in ancestral *Brucella*, and then lost after the divergence of the *B. suis *clade, of *B. neotomae*, of *B. microti *and prior to the divergence of *B. abortus *and *B. melitensis, B. ovis *and *Brucella *sp. strain NVSL 07-2026, and finally marine mammal *Brucella *species. An interesting exception is found in *B. abortus *B3196, where this ABC transporter gene is intact. This feature clearly suggests a divergence of the *B. suis *and *B. neotomae *group before that of the other host-associated *Brucella*.

### IS*711 *insertion sequences

**Figure 3:**
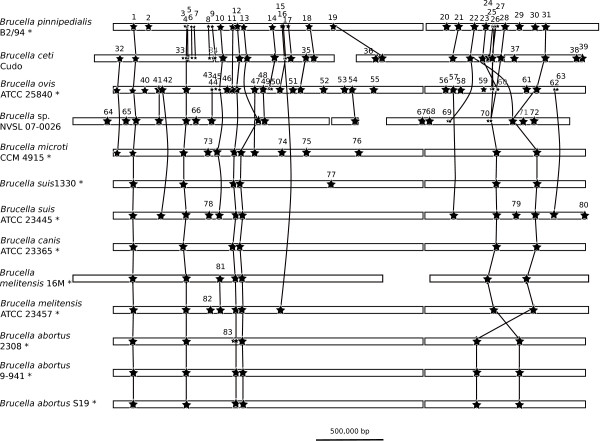
**IS*711 *positions in selected *Brucella *genomes**. Schematic representation of the position of IS*711 *elements in the genome of selected *Brucella *including currently completely assembled *Brucella *(names with an asterisk) and two genomes still in partial form (*B. ceti *Cudo and *Brucella *sp. NVSL 07-0026). IS*711 *positions are noted by a star, some of them in smaller size to increase legibility. Orthologous IS are joined by a line. Each group of connected IS, even isolated ones, is given an identifier which allows to find the corresponding entry in Table S4 which lists the genomic positions of the IS*711 *elements. Chromosomes (or contigs) are depicted as rectangles. The chromosomes of *B. melitensis *16 M are reversed with respect to the original genomic coordinates.

IS*711 *insertion sequences [32] account for a large number of gaps within the complete genome alignment of *B. pinnipedialis *B2/94 and *B. microti *CCM 4915. In total, 19 IS*711 *elements were identified on the large chromosome and 12 on the small chromosome of *B. pinnipedialis*. We looked for the occurrence of those insertion sequences in the available complete genomes of *Brucella *(Methods) and reported their position in Figure 3 and Additional file 5, Table S4. Although it is difficult to analyze transposable repetitive elements in unfinished genomes (as they are often the ones precluding genome assembly) [33], we included two other partial genomes in our analysis, but where an abundant number of IS*711 *elements were observed, *B. ceti *Cudo and *Brucella *sp. strain NVSL 07-0026.

Figure 3 shows that 6 IS*711 *insertion sequences are found in all selected *Brucella *strains (IS*711 *groups numbered 1, 4, 12, 13, 26, and 31). We also observed that 4 IS*711 *elements were uniquely shared by *B. ovis *ATCC 25840 and *Brucella *sp. strain NVSL 07-006 (groups 41, 43, 48 and 54), supporting the grouping of those two species, already noticed when discussing the whole gene tree, and confirmed by the large deletion around the 2.8 kbp fragment discussed previously. Those two species were the likely subjects of an intense IS*711 *transposition activity, with 14 elements being uniquely found in *B. ovis *and 9 being uniquely found in *Brucella *sp. NVSL 07-006. Among the 31 IS*711 *positions found in *B. pinnipedialis *B2/94, 21 are shared with *B. ceti *Cudo among which 12 are found only in *B. pinnipedialis *B2/94 and *B. ceti *Cudo, and absent from all other strains (groups 5-9, 17, 18, 19, 21, 23 and 24).

### Difference in gene content

Comparing orthologous loci (present in both *B. microti *CCM 4915 and *B. pinnipedialis *B2/94 genomes), we found that the number of pseudogenes in the genome of *B. pinnipedialis *B2/94 was larger than that in the genome of *B. microti *CCM 4915 (30 for *B. microti *on chromosome I and 48 for *B. pinnipedialis*, 16 for *B. microti *on chromosome II and 42 for *B. pinnipedialis*). This was also noticed when comparing *B. microti *CCM 4915 and *B. suis *1330, and was attributed to a slower evolution rate in *B. microti*. The genes that are potentially different in both species are reported in Additional file 6, Table S5.

Among the genes altered in *B. pinnipedialis *B2/94, many are components of ABC transporters. Those genes are highlighted in Additional file 6, Table S5. We found 17 genes related to ABC transporters that are impaired, on a total of 90 impaired genes. In the *B. pinnipedialis *genome we identified approximately 249 ABC transporter-related intact genes on a total of 3,342 protein coding genes. This output is highly improbable (p-value = 1.e-4, chi-square test) and strongly suggest that ABC transporters were specifically degraded in *B. pinnipedialis *and more generally in host-associated *Brucella *species. Beside ABC type transport systems, many other genes involved in transport are found impaired in *B. pinnipedialis *B2/94: a CorA family transporter BPI_I592 (ortholog BMI_I558), an EmrB/Qaca family drug resistance transporter BPI_I1098 (ortholog BMI_I1064), the dipeptide transport system permease protein DppC (BPI_I1637, ortholog BMI_I1597), an outer membrane autotransporter BPI_I2072 (ortholog BMI_I2035), a glucose/galactose transporter BPI_II188 (ortholog BMI_II187), a cadmiun-translocating P-type ATPase BPI_II1260 (ortholog BMI_II1204), and finally a putative transport protein BPI_II453 (ortholog BMI_II468).

One of those ABC transporter genes, BPI_I1818, exhibits an interesting feature. A frameshift difference between the *B. pinnipedialis *B2/94 and *B. microti *CCM 4915 sequences merges the membrane and ATP-binding components (BMI_I1778-9) of a thiamin ABC transporter into a single reading frame. A blast (tblastn) search of the BPI_I1818 gene sequence against the nucleotide sequences of the other *Brucella *strains shows that this gene fusion occurred in all marine mammal strains studied, except for *B. ceti *M13/05/1 and M644/93 which represent the distinct dolphin subgroup of strains within *B. ceti *mentioned above.

## Conclusions

### Time of appearance of marine mammal *Brucella *species

It has been suggested [14] that the divergence of species in the genus *Brucella *could have been concomitant with the divergence of their mammalian hosts, 60 millions years (my) ago. However this is inconsistent with the fact that the hosts of *B. ceti *and *B. pinnipedialis *did not diverge at the same time. The ancestors of pinnipeds where carnivores and Higdon *et al*. [34] used molecular data to estimate the split between ursids and pinnipeds to 35.7 ± 2.63 (= mean ± SE) my, and fossil records report early pinnipeds 35 my ago [35]. Cetaceans went back to the sea much earlier, the oldest known cetaceans date back to the Eocene, 55 my ago [36]. If we consider divergence in the 16S rRNA gene sequence, and referring to *B. microti *which has a central position, *B. pinnipedialis, B. melitensis, B. abortus, B. suis *(perhaps with the exception of *B. suis *513, which has 2 (nt) differences, C- > T at position 11 and G- > T at position 1468), *B. ovis, B. canis *have all identical sequences, and *B. neotomae *has 1 bp difference (C- > T at position 541). Using the estimate of 1-2% of change in 16S rRNA sequence per 50 my, 1 bp difference (which really should be considered as a maximal) corresponds to 0.07% change, and a divergence time of 1.75-3.5 my. This time estimate is probably a crude overestimation and recent work [37], using single nucleotide polymorphisms from 13 genomes, showed that most *Brucella *species probably diverged 86,000 to 296,000 years ago. This analysis reveals that the divergence time of *Brucella *sp. found today in marine mammals is totally incompatible with the divergence time of their hosts. A fortuitous contamination of cetaceans and pinnipeds, probably via the food chain, may explain better this transmission of *Brucella *to the marine mammals. This also opens the remote possibility of marine *Brucella *infecting terrestrial mammals.

### On the order of appearance of host-associated *Brucella *species

The analysis of distinctive genomic regions between *B. microti *CCM 4915 and *B. pinnipedialis *B2/94 as well as the study of additional markers reveal the order of appearance of the different *Brucella *species. It is clearly apparent that most of the events following the divergence of *B. microti *from the classical *Brucella *species occurred in a very small amount of time, as if something caused a sudden radiation in this lineage and a subsequent adaptation of the organisms to their hosts. Here we summarize some of the major evolutionary events that highlight the evolutionary history of the genus *Brucella*.

Following the divergence of *B. microti*, the next evolutionary event that we can trace is the 2.6 kbp fragment clear disappearance, which tells us that the two next *Brucella *groups to diverge were *B. suis *and *B. neotomae*. We did not find any good marker in favor of a prior divergence of one versus the other.

Marine mammal *Brucella *species as well as *B. ovis *and *Brucella *sp. NVSL 07-0026 share the presence of a high number of IS*711 *elements, and it has been demonstrated that IS*711 *transposition is still an active process in *B. ovis *and *B. pinnipedialis *[38]. Those insertion elements are much less numerous in *B. melitensis *and *B. abortus *strains and we thus assume that IS*711 *transposition events occurred quite abundantly after the divergence of *B. melitensis*/*B. abortus*. In this group, *B. abortus *strains share a unique feature which is a genomic 600 kbp inversion in the small chromosome, as clearly depicted on Figure 1 of reference [39].

The whole gene tree, where *B. ovis *and *Brucella *sp. NVSL 07-0026 cluster together, the 4 IS*711 *elements positions that they have in common and not shared with *B. pinnipedialis *B2/94 as well as the large deletion that those two species share, in the region surrounding the 2.8 kbp deletion in *B. pinnipedialis*, all those facts support the grouping of *B. ovis *with *Brucella *sp. NVSL 07-0026. The grouping of marine mammal *Brucella *strains that we observe in the tree with early divergence of *B. ceti *M13/05/1 and M644/93 is also reflected by the gene fusion event mentioned earlier. Grouping of *B. pinnipedialis *M292/94 and B2/94 is supported by the loss of the 18 kbp fragment in these two strains.

### The explosive radiation in the genus *Brucella*

There is a clear transition in the genus *Brucella *evolutionary tree. The first *Brucella *discovered were the host-associated *Brucella *species, but more recently, *B. microti *was isolated as the first representative of a fast growing list of free-living *Brucella*. This biochemically highly active bacteria was found to share more phenotypic traits with *Ochrobactrum *than with the host-associated *Brucella *species [6]. We proposed earlier [30] that the transition between a free-living and an host-associated life style could have resulted from the modification in the 23S ribosomal RNA gene sequence with putative effects on the growth rate of the bacteria. A slow growth rate has often been advocated for intra-cellular bacteria, as their survival is often dependent on the survival of their hosts [40]. *Brucella *with this change in 23S structure and its impact on growth rate became suddenly more adapted to an host-associated life style than to a free-living style, and progressively adapted to distinct sets of hosts, giving rise to the main lineages of host-associated *Brucella *species that are encountered today.

## Methods

### Sequencing and origin of sequence data used for comparative work

Genome was assembled from 430,042 paired GS-FLX reads of average length 229, giving approximately a 30× coverage of the genome, and directed sequencing of the remaining gaps was performed using 193 additional Sanger sequencing reactions. The genome sequence is deposited in the complete genome division of GenBank under project ID 41867 and accession numbers CP002078 and CP002079. Genomic sequence data of all *Brucella *strains mentioned in this work can be conveniently downloaded from a unique location at the Pathosystems Resource Integration Center web site [41], or otherwise from the complete genome and whole genome shotgun divisions of GenBank [42]. Origin of the sequence data is listed in Supplementary Table 3. Most sequences originate from the Brucella group project at the Broad Institute, conducted by Davis O'Callaghan, Adrian Whatmore and Renee Tsolis [43] or from the Pathosystems Resource Integration Center of the Virginia Bioinformatics Institute [41].

### Whole gene tree

Whole gene tree was build using gene sequences from all available *Brucella *as well as *Ochrobactrum intermedium*. The procedure used to build the tree is similar to that reported in [30]. Briefly, 1125 orthologous genes from 39 *Brucella *plus genes from *O. intermedium *were used. Orthologous genes were selected using the following procedure. A file containing nucleotide gene sequences for the selected organisms was compared to itself using blastn [44] (parameters: -b 100 -v 100 -F F -e 1.e-20). The resulting output file was subjected to clustering using the Markov chain clustering algorithm [45]. In the resulting cluster list, we selected the clusters with only one unique member per species. Genes from each cluster were then aligned using MUSCLE [46] (default parameters). The following alignments were concatenated, resulting in an alignment of 40 sequences, with 1,078,083 positions, cleaned with Gblock [47] (default parameters), which reduced it to 945,578 positions. From this multiple alignment, on which 767,738 sites without polymorphisms and 2189 distinct patterns were found, a tree was inferred by maximum likelihood using PhyML [48], with 100 bootstrap replicates.

### Unshared genome segments, tracing segments history

The nucleotide sequence of the genome fragment coming either from *B. pinnipedialis *B2/94 or *B. microti *CCM 4915 was used as a query for blastn search (e-value = 1.e-100, no filter) against the nucleotide sequences of the remaining *Brucella*. For each target genome, the number of distinct nucleotide positions that had a hit was recorded in Additional file 3, Table S3. Presence or absence of a given fragment is represented in Figure 1 alongside the whole gene tree. The history of those genome segments was subsequently traced using the Mesquite software package [49] on cladograms reflecting the topology obtained from the whole gene tree (Figure 1). Character history was computed using parsimony analysis, with presence or absence of a genome segment treated as a discrete category character.

### Across genomes identification of IS*711 *element positions

A database containing the complete genomes of the *Brucella *species under study was compiled. We searched this database for the occurrence of IS*711 *using the nucleotide sequence of one element from *B. pinnipedialis *as query, using the blastn program (parameters: -b 1000 -v 1000, limiting to hits with a score of 500). We extracted the corresponding segments, adding 500 nucleotides of context on both side. We then masked this file so that the IS*711 *sequence itself was replaced by × in the sequence. A subsequently blastn search of this file against itself allowed us to recover the orthologous IS*711 *positions along the different *Brucella *genomes (listed in Additional file 5, Table S4), used for the representation in Figure 3.

## Authors' contributions

SA participated in the design of the study, performed genome assembly, annotation and comparisons with other *Brucella *and wrote the manuscript. ML participated in genome annotation and analyzed the data. JMC participated in the design of the study, analyzed data and wrote the manuscript. MZ and AC designed the study, coordinated the research, analyzed data and wrote the manuscript. All authors read and approved the final manuscript.

## Supplementary Material

Additional file 1 **Indels in *B. pinnipedialis *B2/94 and *B. microti *large chromosome alignment**. List of insertions and deletions (indels) in the complete genome alignment of *B. pinnipedialis *B2/94 and *B. microti *CCM 4915, large chromosome. Click here for file

Additional file 2 **Indels in *B. pinnipedialis *B2/94 and *B. microti *small chromosome alignment**. List of insertions and deletions (indels) in the complete genome alignment of *B. pinnipedialis *B2/94 and *B. microti *CCM 4915, small chromosome. Click here for file

Additional file 3 **Occurrences of the largest genome fragments unshared between *B. pinnipedialis *B2/94 and *B. microti *CCM 4915 in selected *Brucella *genomes**. Occurrences of the largest genome fragments unshared between *B. pinnipedialis *B2/94 and *B. microti *CCM 4915 in selected *Brucella *genomes. For each organism, the number of positions in its genome that are found similar to the original fragment is reported. The column "Origin of sequence data" indicates "GenBank" for sequences deposited in the complete genome division of GenBank, "Patric" if the sequence originates from the Pathosystems Resource Integration Center of the Virginia Bioinformatics Institute, and "Broad" if he sequence originates from the Brucella genome sequencing project at the Broad Institute. Click here for file

Additional file 4 **Evolutionary history of the unshared sequence fragments along the *Brucella evolutionary *tree**. Evolutionary history of the unshared sequence fragments along the *Brucella evolutionary *tree. The tree is represented as a cladogram with the same topology as that of the whole gene tree (Figure 1). Plots are generated using Mesquite. Click here for file

Additional file 5 **Position of IS*711 *insertion sequences in the genome of completely sequences *Brucella, B. ceti *Cudo and *Brucella *sp. NVSL 07-0026**. Position of IS*711 *insertion sequences in the genome of completely sequences *Brucella, B. ceti *Cudo and *Brucella *sp. NVSL 07-0026. Each IS*711 *element position as well as the accession number of the genome segment to which it belongs are listed in the Table. Clusters of IS*711 *elements are identified by a number allowing to locate them in Figure 3. Click here for file

Additional file 6 **List of genes showing a large change between *B. pinnipedialis *B2/94 and *B. microti *CCM 4915**. List of genes showing a large change between *B. pinnipedialis *B2/94 and *B. microti *CCM 4915. ABC transporter genes are indicated. Click here for file
