# Supplementary material for: The genome sequence of Brucella pinnipedialis B2/94 sheds light on the evolutionary history of the genus Brucella
Source: BMC Evol Biol. 2011 Jul 11;11:200. doi: 10.1186/1471-2148-11-200 (PMC3146883; doi:10.1186/1471-2148-11-200)
Supplement: Additional file 1 — Indels in B. pinnipedialis B2/94 and B. microti large chromosome alignment. List of insertions and deletions (indels) in the complete genome alignment of B. pinnipedialis B2/94 and B. microti CCM 4915, large chromosome. [file 1471-2148-11-200-S1.DOC]

| **Indels in large chromosome alignment of *B. pinnipedialis* and *B. microti*** | | | | | | |
| --- | --- | --- | --- | --- | --- | --- |
| **Coordinates *B. microti*** | | **Coordinates *B. pinnipedialis*** | | **Indel *B. microti*** | **Indel *B. pinnipedialis*** | **Comment** |
| 208 | -> | 209 | 209 |  | A | Intergenic |
| 33557 | 34400 | -> | 33557 | GGCGTGTCTGCATTTAACGTAACCAGATCATAGCGCATGCGAGATGGACGAAACCCATGAATGCGGTCAATGTTTTCTCGCATCGCAGCGCAATACGACGATAGCGTTTCAACTTGTTAAAAAAGCATTCAATCTGATGGCGTTCCTTGTACAGCCTCCAGTCGATTGTTGGGACACTGGAACGTGTTGGATTGACCTTGATCTGAGCCGTTGCCTTGAGATTGCTGGCAATGAAGGCCCTTAAGTGATCGGCATCATAGGCTGCATCAGCAATGACATGCCCCACACCCTTCAAGCCGGATAGAAGGCTTGAAGCTTGCGGACAGTCACCATAATGGCCGGGTGTTGGCTTTATTCGCAGCGGTAGGCCGATAGCATCGACAACAGCATGCAGCTTGGTCGTCAATCCACCGCGCGAGCGACCGATGCAGGCAGCTTCAGCCCCCCTTTTGCGCCCGCCGCATCTGCGTGGACTTTCGATATGGTGCTATCAATGAGGACATATTCAAAGTCCGGCGTATCAGCCAGGGCATGGAAAAGCCTTTCCCATACACCGGCGTGCGACCAGCGCCGAAAGCGGGCATGAACCGCTGTCCATTTGCCGAAGGTCGCAGGCAGATCGCGCCAGTGCGCTGCATTGGCAGCCATCCACAAGATGGCGTCGACAAATAATCGGTTATCGACGCCACTGCGGCCGGGCGTACCAACTCGCCCCGGAAGATATGCTTCGATCCGGTTCCATTGCTCATCTGTAAGGCTTCGTCTGCTCACGGCTGTTCTCCTTCAACAACCTTGAATCAGAATTTCATGCAAAAGGGAATCCTTGAATGCAGACAAGCCCTAG |  | IS711 in B. microti |
| 39461 | -> | 38619 | 38619 |  | A | Frameshift in BPI_I34, orthologous gene BMI_I36 is pseudogene |
| 48188 | 48276 | -> | 47345 | CAGCAGATGATCCGCGAAGCGCGCGCAAGCCTGCGCAAGTCTTCGCCCATCGATTTCAGCCAGTGGGCGAACGATATCATCTGCACCGT |  | Fragment deleted from BPI_I43, cyoA |
| 49023 | -> | 48093 | 48095 |  | CCA | 1 aa inserted in BPI_I44, cyoB |
| 51534 | -> | 50607 | 50616 |  | TCTCTCCCCC | Change in hypothetical protein BPI_I47/BMI_I49 |
| 53990 | 53990 | -> | 53071 | G |  | frameshift in BPI_I49, bacterial surface antigene which is a pseudogene (orth: BMI_I51) |
| 63696 | 63783 | -> | 62776 | CTGCCCTACTGCCCTACTGCCCTACTGCCCTACTGCCCTACTGCCCTACTGCCCTACTGCCCTACTGCCCTACTGCCCTACTGCCCTA |  | Fragment deleted from BPI_I58, mrp-related protein (orth: BMI_I60) |
| 80504 | 80828 | -> | 79496 | TTGAAACGCAGCATCACCAATGTCGGTGCAGGGCGTGTGAGCAAGGACTCGACGGATGCTGTCAACGGCAGCCAGCTTTACGCCGTTGCGGAACAGGCCACGTTAGGCTGGAACCTGACGGCAAATGGAACGGATAAAAGCAGGGTTTCACCGGGCGACACGGTTGACCTGAGCAATAGCGATGGCAATTTCGTTATTGGCAAGCATGGCACTGGCGTTACGTTTAACCTTGCACCAGATTTGAAAGTAACGAGCCTTGTAGCGGGTAACACCTTTCTGGATACGAACGGACTTGTGATTACCGGCGGTCCGAGCATGACAGTGT |  | Fragment deleted from BPI_I73 (pseudogene, orth: BMI_I75), outer membrane protein. |
| 80835 | 80838 | -> | 79502 | TTGA |  | same as above |
| 80845 | 80854 | -> | 79508 | TCATCTGAAA |  | same as above |
| 82474 | 82474 | -> | 81127 | T |  | same as above |
| 88608 | -> | 87262 | 87262 |  | T | Intergenic |
| 106738 | 106738 | -> | 105391 | T |  | Intergenic |
| 107788 | 107788 | -> | 106440 | A |  | Frameshift in BPI_I103, pseudogene of response regulator, receiver domain (orth: BMI_I105) |
| 110240 | -> | 108893 | 108893 |  | T | Intergenic |
| 122123 | 122124 | -> | 120775 | CG |  | Frameshift in BPI_I112, cyclic beta 1-2 glucan synthetase pseudogene (orth: BMI_I114) |
| 139752 | 139752 | -> | 138403 | G |  | Frameshift in BPI_I125, ATP-dependent RNA helicase, DEAD/DEAH box family (orth: BMI_I127) |
| 139600 | -> | 138252 | 138252 |  | T | Intergenic |
| 144047 | 144047 | -> | 142697 | A |  | Intergenic |
| 152279 | -> | 150930 | 150930 |  | G | Frameshift in BPI_I136, IS711 transposase orfB pseudogene, (orth: BMI_I138) |
| 163290 | 163290 | -> | 161940 | A |  | Frameshift in BPI_I147, malate dehydrogenase (oxaloacetate-decarboxylating) (NADP+), phosphate acetyltransferase pseudogene (orth: BMI_I149) |
| 170077 | 170279 | -> | 168726 | CTTCCTTTACCTGGCCGACCTTCGGGTGTTCGGACCGGTGTTCGGGGCGCGAGCCGCCGCTTCTGGAGAATTCGTCGTTACGGCTCATTTGGCTACCCAGCGGCTGAACATGGATGTGGAAAGCGCGCGCCCTCCCGACTGATCGGCGGAGCGTTCGCGCAGGATGAGTTCGCCTGATTCGACCGTGCCGCCAAGGCCGGTGA |  | Fragment deleted from BPI_I152. The gene is shorter that its counterpart in other Brucella. (orth: BMI_I154). This deletion also impair BPI_I151, becoming a pseudogene (orth: BMI_I153). |
| 206553 | -> | 205001 | 205016 |  | TTTGAGGACCAGCTTG | Frameshift in BPI_I195, cysNC, bifunctional sulfate adenylyltransferase subunit 1/adenylylsulfate kinase protein (orth: BMI_I197) |
| 216742 | 216799 | -> | 215204 | AAAAGTGGGAACCGGTTTTCCGACCACGACATGCGTATAGGCAAAAAGCATGTCGCGG |  | Intergenic |
| 260784 | -> | 259190 | 280902 |  | CATCTTATTGAAGTTTCAAGGTAATTCCACAAGTTTC up to TTTACTGAAAAATGGTGCCCGGAGGCGG | 21 kbp fragment absent from B. microti, encoding genes BPI_I248 to BPI_I279 |
| 269733 | -> | 289852 | 289862 |  | AGTTCGCGCTC | change gene BPI_I292 (orth: BMI_I263), hypothetical protein |
| 271786 | 271786 | -> | 291914 | C |  | Frameshift in BPI_I295 pseudogene (orth: BMI_I266), hypothetical protein |
| 273035 | -> | 293164 | 293167 |  | GAAC | Intergenic |
| 273184 | 273192 | -> | 293315 | TTCCGAATC |  | In gene BPI_I299, a gene is annotated on the other direction in B. microti (BMI_I267). Both are probably overpredictions |
| 273292 | 273292 | -> | 293414 | C |  | same as above |
| 273364 | -> | 293487 | 293489 |  | TCG | same as above |
| 273381 | -> | 293507 | 293519 |  | TCTTCATAATCAG | same as above |
| 273406 | 273406 | -> | 293543 | T |  | same as above |
| 273423 | -> | 293561 | 293577 |  | TTGTGCCGTCTCCCTTG | same as above |
| 273438 | -> | 293593 | 293601 |  | GTCCACAAG | same as above |
| 273462 | 273462 | -> | 293624 | C |  | Intergenic |
| 273473 | 273477 | -> | 293634 | CGAGG |  | Intergenic |
| 273501 | -> | 293658 | 293662 |  | AACCC | Intergenic |
| 273526 | 273535 | -> | 293686 | TTCGCTCTCC |  | Intergenic |
| 273570 | -> | 293722 | 293731 |  | CTCCATTATG | Intergenic |
| 273578 | -> | 293740 | 293775 |  | GAGAGACATCCCAGCCGTCCGGCACACCGCCATCCG | Intergenic |
| 273599 | 273599 | -> | 293795 | T |  | Intergenic |
| 273858 | 273858 | -> | 294053 | A |  | Intergenic |
| 297063 | 297164 | -> | 317257 | AAAAGTGCAAGCGGTTTTCGGAGCAAGATCATGCTTAAACAAAGAGATAGAGCGGTTCCAACGATTCCGTTTTAACCGGAACCGCTCTGGAGCATGATCCCG |  | Intergenic |
| 323493 | -> | 343587 | 343587 |  | G | Frameshift in BPI_I345. BMI_I316 is a pseudogene of MATE efflux family protein |
| 346201 | 346202 | -> | 366294 | CA |  | Frameshift in BPI_I367 causes small difference in the beginning of the gene with respect to BMI_I340, saccharopine dehydrogenase |
| 351589 | -> | 371682 | 371682 |  | C | In gene BPI_I375. Frameshift causes BMI_I346 to become a pseudogene, sensor histidine kinase. |
| 366804 | 366804 | -> | 386896 | C |  | Changes end of membrane spanning protein BPI_I387 (orth: BMI_I358) |
| 380619 | -> | 400712 | 400750 |  | TGCGGCTATCCATGAGGTCGGCAGCCTTGTTCAACAGTG | in-frame change in BPI_I401, succinate semialdehyde dehydrogenase (orth: BMI_I372) |
| 391786 | 391847 | -> | 411916 | TCGAGCCAAAAGTGCGTAGCGGTTTTGCGTTGGATAATGCGACCGGCAAAAAGTGAGCATTT |  | Intergenic |
| 399559 | 399983 | -> | 419627 | GTATTGCCGCGAACTGGCGTGGAGCCACCCTGCATGAAAGCCTTGTCCTGAGCGGCCATATTTGCCGGCTGGCCGACATAGGGGTTTTCGGCATAGGCGGCACCGGCGCTAAGAGCAACGGCAAGAGCGGCGAGAGCAAATTTCTTCATTTTTCTAATCCTTCTGGTCGCCCCTTTGGATGGCATAGAAGCCTTCCAGGGCCTCCCGGGTGGCAAACATGCCATCCATCGGGGAGATAATTGTGTGAGCCAGAAAATCTGGGAGGAAACATCCGGCTCATAGGTCAGTAAAGTGGGCTAATGCCCTGCATGGTCAGTCTGAAACGGCCTTAGTTCTGGTAGGACGAAGGGGAAGCATCGCCGAAACGATGGGCCGAACCATCCTGATAAACCGGAGCCTTGATCGAAGCCGGAGCCGTATAGTCG |  | In intergenic region between BPI_I421 and BPI_I424. Hypothetical proteins BMI_I393 and BMI_I393 are annotated in B. microti in this region. |
| 410158 | 410383 | -> | 429801 | GCTCGAACTCTTTTCCGAAGAAATCCCCGCTCTGCCGTCCGCCCGCAGCGAAGCGAGGACGGACAAATTGAATAATGTGCATGCGGAAGGCGTACGCTGATGCCTGATTTGTTGCTCGAACTCTTTTCCGAAGAAATCCCCGCTCTGCCGTCCGCCCGCAGCGAAGCGAGGACGGACAAATTGAATAATGTGCATGCGGAAGGCGTACGCTGATGCCTGATTTGTT |  | Intergenic |
| 416710 | -> | 436129 | 436143 |  | CGGTGCGCACCGGCC | In frame change in BPI_I441, hypothetical protein (orth:BMI_I414) |
| 428825 | -> | 448260 | 448260 |  | A | Intergenic |
| 440952 | 440975 | -> | 460386 | GCCCTACTGCCCTACTGCCCTACT |  | Intergenic |
| 456820 | 456820 | -> | 476230 | A |  | Frameshift in gene BPI_I482, hypothetical protein, pseudogene BMI_I455 in B. microti. |
| 470923 | 470923 | -> | 490332 | G |  | Frameshift in BPI_I498, protoheme IX farnesyltransferase pseudogene (orth: BMI_I471) |
| 479789 | 479804 | -> | 499197 | CTGCCCTACTGCCCTA |  | Intergenic |
| 486418 | 486477 | -> | 505810 | GGTTTTGCGTTGGATAATGCGACCAGGAAAGGTTTGCATTTCGAGCCAAAAGTGCGAAGC |  | Change in gene BPI_I514, shorter than BMI_I487. Both are annotated as hypothetical protein. |
| 505982 | 505982 | -> | 525314 | A |  | Frameshift in BPI_I530 with respect to B. microti pseudogene BMI_I503, polysaccharide deacetylase. |
| 508029 | 508029 | -> | 527360 | C |  | Frameshift in BPI_I532 corresponding to B. microti pseudogene BMI_I506, metallo-beta-lactamase family protein. |
| 506759 | -> | 526092 | 526092 |  | T | Intergenic |
| 517297 | -> | 536629 | 537472 |  | GGCGTGTCTGCATTCAACGCAACCAGATCATAGCGCATGCGAGATGGACGAAGCCCATGAATGCGGTCAATGTTTTCTCGCATCGCAGCGCAATACGACGATAGCGTTTCAACTTGTTAAAAAAGCATTCAATCTGATGGCGTTCCTTGTACAGCCTCCAGTCGATTGTTGGGACACTGGAACGTGTTGGATTGGCCTTGATCTGAGCCGTTGCCTTGAGATTGCTGGCAATGAAGGCCCTTAAGTGATCGGCATCATAGGCCGCATCAGCAATGACATGCCCCACACCCTCTAAGCCGGATAGAAGGCTTGAAGCTTGCGGACAGTCACCATAATGGCCGGGTGTTGGCTTTATTCGCAGCGGTAGGCCGATAGCATCGACAACAGCATGCAGCTTGGTCGTCAATCCACCGCGCGAGCGACCGATGCAGGCAGCTTCAGCCCCCCTTTTGCGCCCGCCGCATCTGCGTGGACTTTCGATATGGTGCTATCAATGAGGACATATTCAAAGTCCGGCGTATCAGCCAGGGCATGGAAAAGCCTTTCCCATACACCGGCGTGCGACCAGCGCCGAAAGCGGGCATGAACCGCTGTCCATTTGCCGAAGGTCGCAGGCAGATCGCGCCAGTGCGCTGCATTGGCAGCCATCCACAAGATGGCGTCGACAAATAATCGGTTATCGACGCCACTGCGGCCGGGCGTACCAACTCGCCCCGGAAGATATGCTTCGATCCGGTTCCATTGCTCATCTGTAAGGCTTCGTCTGCTCACGGCTGTTCTCCTTTAACAACCTTGAATCAGAATTTCGTACAAAAGGGAATCCTTGAATGCAGACAAGCCCTAG | IS711 family transposase |
| 526964 | -> | 547140 | 547140 |  | T | This and following framshift produce a small change in the sequence of IS711 insertion sequence transposase OrfB |
| 526988 | 526988 | -> | 547163 | T |  | see above |
| 532605 | -> | 552781 | 553624 |  | GGCGTGTCTGCATTCAACGCAACCAGATCATAGCGCATGCGAGATGGACGAAGCCCATGAATGCGGTCAATGTTTTCTCGCATCGCAGCGCAATACGACGATAGCGTTTCAACTTGTTAAAAAAGCATTCAATCTGATGGCGTTCCTTGTACAGCCTCCAGTCGATTGTTGGGACACTGGAACGTGTTGGATTGGCCTTGATCTGAGCCGTTGCCTTGAGATTGCTGGCAATGAAGGCCCTTAAGTGATCGGCATCATAGGCCGCATCAGCAATGACATGCCCCACACCCTCTAAGCCGGATAGAAGGCTTGAAGCTTGCGGACAGTCACCATAATGGCCGGGTGTTGGCTTTATTCGCAGCGGTAGGCCGATAGCATCGACAACAGCATGCAGCTTGGTCGTCAATCCACCGCGCGAGCGACCGATGCAGGCAGCTTCAGCCCCCCTTTTGCGCCCGCCGCATCTGCGTGGACTTTCGATATGGTGCTATCAATGAGGACATATTCAAAGTCCGGCGTATCAGCCAGGGCATGGAAAAGCCTTTCCCATACACCGGCGTGCGACCAGCGCCGAAAGCGGGCATGAACCGCTGTCCATTTGCCGAAGGTCGCAGGCAGATCGCGCCAGTGCGCTGCATTGGCAGCCATCCACAAGATGGCGTCGACAAATAATCGGTTATCGACGCCACTGCGGCCGGGCGTACCAACTCGCCCCGGAAGATATGCTTCGATCCGGTTCCATTGCTCATCTGTAAGGCTTCGTCTGCTCAAGGCTGTTCTCCTTTAACAACCTTGAATCAGAATTTCGTACAAAAGGGAATCCTTGAATGCAGACAAGCCCTAG | IS711 family transposase in the sequence of gene ManB, making it a pseudogene in B. pinnipedialis (BPI_I566). (orth: BMI_I536) |
| 538270 | 538270 | -> | 559288 | C |  | Frameshift making BPI_I575, ribose ABC transporter, ATP-binding protein a pseudogene (orth: BMI_I541) |
| 549031 | 549031 | -> | 570048 | T |  | Intergenic |
| 553612 | -> | 574630 | 574630 |  | C | Frameshift causing BPI_I588, L-asparaginase II protein to be a pseudogene in B. microti. (orth: BMI_I554) |
| 556063 | 556063 | -> | 577080 | G |  | Frameshift, BPI_I592 pseudogene transporter, CorA family (orth: BMI_I558). |
| 559398 | -> | 580416 | 581261 |  | TAGGGCGTGTCTGCATTTAACGTAACCAGATCATAGCGCATGCGAGATGGACGAAACCCATGAATGCGGTCAATGTTTTCTCGCATCGCAGCGCAATACGACGATAGCGTTTCAACTTGTTAAAAAAGCATTCAATCTGATGGCGTTCCTTGTACAGCCTCCAGTCGATTGTTGGGACACTGGAACGTGTTGGATTGACCTTGATCTGAGCCGTTGCCTTGAGATTGCTGGCAATGAAGGCCCTTAAGTGATCGGCATCATAGGCCGCATCAGCAATGACATGCCCCACACCCTCTAAGCCGGATAGAAGGCTTGAAGCTTGCGGACAGTCACCATAATGGCCGGGTGTTGGCTTTATTCGCAGCGGTAGGCCGATAGCATCGACAACAGCATGCAGCTTGGTCGTCAATCCACCGCGCGAGCGACCGATGCAGGCAGCTTCAGCCCCCCTTTTGCGCCCGCCGCATCTGCGTGGACTTTCGATATGGTGCTATCAATGAGGACATATTCAAAGTCCGGCGTATCAGCCAGGGCATGGAAAAGCCTTTCCCATACACCGGCGTGCGACCAGCGCCGAAAGCGGGCATGAACCGCTGTCCATTTGCCGAAGGTCGCAGGCAGATCGCGCCAGTGCGCTGCATTGGCAGCCATCCACAAGATGGCGTCGACAAATAAATCGAGTTATCGACGCCACTGCGGCCGGGCGTACCAACTCGCCCCGGAAGATATGCTTCGATCCGGTTCCATTGCTCATCTGTAAGGCTTCGTCTGCTCAAGGCTGTTCTCCTTCAACAACCTTGAATCAGAATTTCATGCAAAAGGGAATCCTTGAATGCAGACAAGCCC | IS711 family transposase |
| 561081 | -> | 582945 | 582952 |  | GGGAATAG | Intergenic |
| 569398 | -> | 591270 | 591270 |  | G | Small change at the end of BPI_I608 with respect to ortholog BMI_I572 |
| 576588 | -> | 598461 | 599304 |  | GGGCGTGTCTGCATTCAACGCAACCAGATCATAGCGCATGCGAGATGGACGAAGCCCATGAATGCGGTCAATGTTTTCTCGCATCGCAGCGCAATACGACGATAGCGTTTCAACTTGTTAAAAAAGCATTCAATCTGATGGCGTTCCTTGTACAGCCTCCAGTCGATTGTTGGGACACTGGAACGTGTTGGATTGGCCTTGATCTGAGCCGTTGCCTTGAGATTGCTGGCAATGAAGGCCCTTAAGTGATCGGCATCATAGGCCGCATCAGCAATGACATGCCCCACACCCTCTAAGCCGGATAGAAGGCTTGAAGCTTGCGGACAGTCACCATAATGGCCGGGTGTTGGCTTTATTCGCAGCGGTAGGCCGATAGCATCGACAACAGCATGCAGCTTGGTCGTCAATCCACCGCGCGAGCGACCGATGCAGGCAGCTTCAGCCCCCCTTTTGCGCCCGCCGCATCTGCGTGGACTTTCGATATGGTGCTATCAATGAGGACATATTCAAAGTCCGGCGTATCAGCCAGGGCATGGAAAAGCCTTTCCCATACACCGGCGTGCGACCAGCGCCGAAAGCGGGCATGAACCGCTGTCCATTTGCCGAAGGTCGCAGGCAGATCGCGCCAGTGCGCTGCATTGGCAGCCATCCACAAGATGGCGTCGACAAATAATCGGTTATCGACGCCACTGCGGCCGGGCGTACCAACTCGCCCCGGAAGATATGCTTCGATCCGGTTCCATTGCTCATCTGTAAGGCTTCGTCTGCTCACGGCTGTTCTCCTTTAACAACCTTGAATCAGAATTTCGTACAAAAGGGAATCCTTGAATGCAGACAAGCCCTA | IS711 family transposase |
| 585699 | -> | 608416 | 608416 |  | C | Intergenic |
| 587992 | 587992 | -> | 610708 | C |  | Frameshift in BPI_I633, hypothetical protein pseudogene (orth: BMI_I595) |
| 613912 | 613913 | -> | 636627 | CT |  | Intergenic |
| 629436 | -> | 652151 | 652151 |  | C | Frameshift in BPI_I669, hypothetical protein pseudogene (orth: BMI_I632) |
| 634790 | 634790 | -> | 657504 | T |  | Intergenic |
| 641796 | -> | 664511 | 664565 |  | TTTCGGATAAGATGCGCGCAAGGAAAGATCTGTATCCCGAAAAGTGCGAAGCGGT | Intergenic |
| 648732 | 648732 | -> | 671500 | A |  | Frameshift in BPI_I690, coproporphyrinogen III oxidase pseudogene (orth: BMI_I654) |
| 665741 | 665874 | -> | 688508 | GAGAACAATGATGGCACGCGAGCGCATTTATATCTACGACACGACCTCGGTGGATGGGCAGCAGACGTTACCCGCCCGCCCGCAGCCGCGAAGCGAGCGAGGACGGGCAAAGGCAAAAGCGGGCGTTTGCCTGG |  | Intergenic |
| 668007 | -> | 690642 | 690642 |  | G | Intergenic |
| 685861 | 685862 | -> | 709339 | CC |  | Intergenic |
| 685030 | -> | 707666 | 708509 |  | GGGCGTGTCTGCATTCAACGCAACCAGATCATAGCGCATGCGAGATGGACGAAGCCCATGAATGCGGTCAATGTTTTCTCGCATCGCAGCGCAATACGACGATAGCGTTTCAACTTGTTAAAAAAGCATTCAATCTGATGGCGTTCCTTGTACAGCCTCCAGTCGATTGTTGGGACACTGGAACGTGTTGGATTGGCCTTGATCTGAGCCGTTGCCTTGAGATTGCTGGCAATGAAGGCCCTTAAGTGATCGGCATCATAGGCCGCATCAGCAATGACATGCCCCACACCCTCTAAGCCGGATAGAAGGCTTGAAGCTTGCGGACAGTCACCATAATGGCCGGGTGTTGGCTTTATTCGCAGCGGTAGGCCGATAGCATCGACAACAGCATGCAGCTTGGTCGTCAATCCACCGCGCGAGCGACCGATGCAGGCAGCTTCAGCCCCCCTTTTGCGCCCGCCGCATCTGCGTGGACTTTCGATATGGTGCTATCAATGAGGACATATTCAAAGTCCGGCGTATCAGCCAGGGCATGGAAAAGCCTTTCCCATACACCGGCGTGCGACCAGCGCCGAAAGCGGGCATGAACCGCTGTCCATTTGCCGAAGGTCGCAGGCAGATCGCGCCAGTGCGCTGCATTGGCAGCCATCCACAAGATGGCGTCGACAAATAATCGGTTATCGACGCCACTGCGGCCGGGCGTACCAACTCGCCCCGGAAGATATGCTTCGATCCGGTTCCATTGCTCATCTGTAAGGCTTCGTCTGCTCACGGCTGTTCTCCTTTAACAACCTTGAATCAGAATTTCGTACAAAAGGGAATCCTTGAATGCAGACAAGCCCTA | IS711 family transposase |
| 708832 | 709676 | -> | 732309 | GGCGTGTCTGCATTTAACGTAACCAGATCATAGCGCATGCGAGATGGACGAAACCCATGAATGCGGTCAATGTTTTCTCGCATCGCAGCGCAATACGACGATAGCGTTTCAACTTGTTAAAAAAGCATTCAATCTGATGGCGTTCCTTGTACAGCCTCCAGTCGATTGTTGGGACACTGGAACGTGTTGGATTGGCCTTGGATCTGAGCCGTTGCCTTGAGATCGCTGGCAATGAAGGCCCTTAAGTGATCGGCATCATGGGCCGCGTCAGCAATGACATGTCCCACACCCTTTAAGCCGGATAGAAGGCTTGAAGCTTGCGGACAGTCACCATAATGGCCGGGTGTTGGCTTTATTCGCAGCGGTAGGCCGATAGCATCGACAACAGCATGCAGCTTGGTCGTCAATCCACCGCGCGAGCGACCGATGCAGGCAGCTTCAGCCCCCCTTTTGCGCCCGCCGCATCTGCGTGGACTTTCGATATGGTGCTATCAATGAGGACATATTCAAAGTCCGGCGTATCAGCCAGGGCATGGAAAAGCCTTTCCCATACACCGGCGTGCGACCAGCGCCGAAAGCGGGCATGAACCGCTGTCCATTTGCCGAAGGTCGCAGGCAGATCGCGCCAGTGCGCTGCATTGGCAGCCATCCACAAGATGGCGTCGACAAATAATCGGTTATCGACGCCACTGCGGCCGGGCGTACCAACTCGCCCCGGAAGATATGCTTCGATCCGGTTCCATTGCTCATCTGTAAGGCTTCGTCTGCTCACGGCTGTTCTCCTTCAACAACCTTGAATCAGAATTTCATGCAAAAGGGAATCCTTGAATGCAGACAAGCCCTAG |  | IS711 family transposase |
| 704865 | -> | 728343 | 728343 |  | C | Intergenic |
| 714967 | -> | 737601 | 738444 |  | GGGCGTGTCTGCATTCAACGCAACCAGATCATAGCGCATGCGAGATGGACGAAGCCCATGAATGCGGTCAATGTTTTCTCGCATCGCAGCGCAATACGACGATAGCGTTTCAACTTGTTAAAAAAGCATTCAATCTGATGGCGTTCCTTGTACAGCCTCCAGTCGATTGTTGGGACACTGGAACGTGTTGGATTGGCCTTGATCTGAGCCGTTGCCTTGAGATTGCTGGCAATGAAGGCCCTTAAGTGATCGGCATCATAGGCCGCATCAGCAATGACATGCCCCACACCCTCTAAGCCGGATAGAAGGCTTGAAGCTTGCGGACAGTCACCATAATGGCCGGGTGTTGGCTTTATTCGCAGCGGTAGGCCGATAGCATCGACAACAGCATGCAGCTTGGTCGTCAATCCACCGCGCGAGCGACCGATGCAGGCAGCTTCAGCCCCCCTTTTGCGCCCGCCGCATCTGCGTGGACTTTCGATATGGTGCTATCAATGAGGACATATTCAAAGTCCGGCGTATCAGCCAGGGCATGGAAAAGCCTTTCCCATACACCGGCGTGCGACCAGCGCCGAAAGCGGGCATGAACCGCTGTCCATTTGCCGAAGGTCGCAGGCAGATCGCGCCAGTGCGCTGCATTGGCAGCCATCCACAAGATGGCGTCGACAAATAATCGGTTATCGACGCCACTGCGGCCGGGCGTACCAACTCGCCCCGGAAGATATGCTTCGATCCGGTTCCATTGCTCATCTGTAAGGCTTCGTCTGCTCACGGCTGTTCTCCTTTAACAACCTTGAATCAGAATTTCGTACAAAAGGGAATCCTTGAATGCAGACAAGCCCTA | IS711 family transposase |
| 721916 | 721916 | -> | 745392 | A |  | Frameshift in gene BPI_I770, hypothetical protein. Ortholog BMI_I731 is a pseudogene. |
| 731341 | 731341 | -> | 754816 | G |  | Frameshift in BPI_I780, amino acid ABC transporter, permease protein pseudogene (orth: BMI_I741). |
| 755515 | 755529 | -> | 778989 | CCAATATTGAAATTG |  | Intergenic |
| 779960 | 779962 | -> | 803419 | GGG |  | In frame change in BPI_I834 (orth: BMI_I795), hypothetical protein. |
| 782017 | -> | 805475 | 805475 |  | G | Intergenic |
| 810878 | 810878 | -> | 834335 | A |  | Intergenic |
| 813100 | 813100 | -> | 836556 | A |  | Intergenic |
| 857368 | -> | 880825 | 880825 |  | T | Frameshift in BPI_I915, beta-hexosaminidase A. B. microti ortholog BMI_I876 is a pseudogene. |
| 863941 | 864005 | -> | 887397 | GGTCGCGGCAAGGCTCCGGTTGCCGACGACAGCGATATTGCTGCCATCCGTTCGGGTTGCATTTC |  | Change in acid phosphatase SurE, pseudogene BPI_I922 (orth: BMI_I883). |
| 864849 | -> | 888242 | 889085 |  | GGGCGTGTCTGCATTCAACGCAACCAGATCATAGCGCATGCGAGATGGACGAAGCCCATGAATGCGGTCAATGTTTTCTCGCATCGCAGCGCAATACGACGATAGCGTTTCAACTTGTTAAAAAAGCATTCAATCTGATGGCGTTCCTTGTACAGCCTCCAGTCGATTGTTGGGACACTGGAACGTGTTGGATTGGCCTTGATCTGAGCCGTTGCCTTGAGATTGCTGGCAATGAAGGCCCTTAAGTGATCGGCATCATAGGCCGCATCAGCAATGACATGCCCCACACCCTCTAAGCCGGATAGAAGGCTTGAAGCTTGCGGACAGTCACCATAATGGCCGGGTGTTGGCTTTATTCGCAGCGGTAGGCCGATAGCATCGACAACAGCATGCAGCTTGGTCGTCAATCCACCGCGCGAGCGACCGATGCAGGCAGCTTCAGCCCCCCTTTTGCGCCCGCCGCATCTGCGTGGACTTTCGATATGGTGCTATCAATGAGGACATATTCAAAGTCCGGCGTATCAGCCAGGGCATGGAAAAGCCTTTCCCATACACCGGCGTGCGACCAGCGCCGAAAGCGGGCATGAACCGCTGTCCATTTGCCGAAGGTCGCAGGCAGATCGCGCCAGTGCGCTGCATTGGCAGCCATCCACAAGATGGCGTCGACAAATAATCGGTTATCGACGCCACTGCGGCCGGGCGTACCAACTCGCCCCGGAAGATATGCTTCGATCCGGTTCCATTGCTCATCTGTAAGGCTTCGTCTGCTCAAGGCTGTTCTCCTTTAACAACCTTGAATCAGAATTTCGTACAAAAGGGAATCCTTGAATGCAGACAAGCCCTA | IS711 family transposase |
| 877158 | 877170 | -> | 901393 | AAGGCCGAAGAAG |  | Change at the end of BPI_I938, trigger factor tig (orth: BMI_I897). |
| 881222 | 881222 | -> | 905444 | G |  | Intergenic |
| 897063 | 897063 | -> | 921284 | C |  | BPI_I959, IS711 transposase OrfB, is pseudogene BMI_I918 is B. microti. |
| 919341 | 919341 | -> | 943561 | G |  | Intergenic |
| 928716 | 931368 | -> | 952935 | TGGTGGTTGATACCCAATCCATC up to AGTTGGCGTGCGTCGTGGAACCCTGCACA |  | 2.6 kbp insert in B. microti, between BPI_I990 and BPI_I993. Due to the presence of intact genes in B. microti, this is clearly a deletion in B. pinnipedialis. |
| 935050 | -> | 956618 | 956618 |  | A | Change in gene BPI_I995 with respect to BMI_I957. Both are hypothetical proteins. |
| 938738 | 938813 | -> | 960305 | AAGCTGTATCCGCGCCGCAAGCCCCTGTACGCGCCCTTCCGCCCGTTCAGCAGCGCGTGACGGTGCAGGGCCCCCA |  | BPI_I998, hypothetical protein, is slighly shorter than its ortholog BMI_I960. |
| 942988 | 942988 | -> | 964479 | C |  | Frameshift in BPI_I1005, site-specific recombinase, phage integrase family pseudogene (orth: BMI_I967) |
| 946130 | 946130 | -> | 967620 | T |  | Frameshift in BPI_1010, outer membrane protein pseudogene (orth: BMI_I972) |
| 949373 | 949373 | -> | 970862 | C |  | Frameshit. BPI_I1017 , IS711 transposase orfB, is pseudogene in B. microti (orth: BMI_I979). |
| 949697 | -> | 971187 | 971187 |  | A | Frameshift. BPI_I1018, hypothetical protein, is pseudogene BMI_I980 in B. microti. |
| 997212 | -> | 1018703 | 1018714 |  | GACCGCAGCGGC | Slight in frame change in BPI_I1065, hypothetical protein (orth: BMI_I1027). |
| 1004626 | 1004626 | -> | 1026127 | G |  | Frameshift in BPI_I1075, hypothetical protein pseudogene (orth: BMI_I1037) |
| 1019998 | -> | 1041500 | 1041500 |  | A | Intergenic |
| 1026187 | 1026187 | -> | 1047688 | C |  | Frameshift in BPI_I1095, ATP-dependent RNA helicase, DEAD/DEAH box family pseudogene (orth: BMI_I1057). |
| 1047382 | 1048225 | -> | 1068882 | GGCGTGTCTGCATTTAACGTAACCAGATCATAGCGCATGCGAGATGGTTGAAACCCATGAATGCGGTCAATGTTTTCTCGCATCGCAGCGCAATACGACGATAGCGTTTCAACTTGTTAAAAAAGCATTCAATCTGATGGCGTTCCTTGTACAGCCTCCAGTCGATTGTTGGGACACTGGAACGTGTTGGATTGACCTTGATCTGAGCCGTTGCCTTGAGATTGCTGGCAATGAAGGCCCTTAAGTGATCGGCATCATAGGCCGCGTCAGCAATGACATGTCCCACACCCTTTAAGCCGGATAGAAGGCTTGAAGCTTGCGGACAGTCACCATAATGGCCGGGTGTTGGCTTTATTCGCAGCGGTAGGCCGATAGCATCGACAACAGCATGCAGCTTGGTCGTCAATCCACCGCGCGAGCGACCGATGCAGGCAGCTTCAGCCCCCCTTTTGCGCCCGCCGCATCTGCGTGGACTTTCGATATGGTGCTATCAATGAGGACATATTCAAAGTCCGGCGTATCAGCCAGGGCATGGAAAAGCCTTTCCCATACACCGGCGTGCGACCAGCGCCGAAAGCGGGCATGAACCGCTGTCCATTTGCCGAAGGTCGCAGGCAGATCGCGCCAGTGCGCTGCATTGGCAGCCATCCACAAGATGGCGTCGACAAATAATCGGTTATCGACGCCACTGCGGCCGGGCGTACCAACTCGCCCCGGAAGATATGCTTCGATCCGGTTCCATTGCTCATCTGTAAGGCTTCGTCTGCTCACGGCTGTTCTCCTTCAACAACCTTGAATCAGAATTTCATGCAAAAGGGAATCCTTGAATGCAGACAAGCCCTAG |  | IS711 family transposase |
| 1055765 | 1055765 | -> | 1076421 | T |  | Intergenic |
| 1056023 | 1056023 | -> | 1076678 | T |  | Intergenic |
| 1056041 | -> | 1076697 | 1076732 |  | CGGATGGCGGTGTGCCGGACGGCTGGGATGTCTCTC | Intergenic |
| 1056049 | -> | 1076741 | 1076750 |  | CATAATGGAG | Intergenic |
| 1056085 | 1056094 | -> | 1076785 | GGAGAGCGAA |  | Intergenic |
| 1056118 | -> | 1076810 | 1076814 |  | GGGTT | Intergenic |
| 1056138 | 1056138 | -> | 1076833 | G |  | Intergenic |
| 1056145 | 1056148 | -> | 1076838 | TCGC |  | Intergenic |
| 1056158 | 1056158 | -> | 1076847 | G |  | Intergenic |
| 1056181 | -> | 1076871 | 1076879 |  | CTTGTGGAC | In hypothetical protein BPI_I1125 |
| 1056196 | -> | 1076895 | 1076911 |  | CAAGGGAGACGGCACAA | In hypothetical protein BPI_I1125 |
| 1056214 | 1056214 | -> | 1076928 | A |  | In hypothetical protein BPI_I1125 |
| 1056251 | -> | 1076966 | 1076980 |  | TCACGGAGAAGGGAG | In hypothetical protein BPI_I1125 |
| 1056257 | -> | 1076987 | 1076987 |  | A | In hypothetical protein BPI_I1125 |
| 1056328 | 1056328 | -> | 1077057 | G |  | In hypothetical protein BPI_I1125 |
| 1056430 | 1056438 | -> | 1077158 | TTCGGAAGA |  | In hypothetical protein BPI_I1125 |
| 1056584 | -> | 1077305 | 1077308 |  | GTTC | In hypothetical protein BPI_I1125 |
| 1057834 | 1057834 | -> | 1078557 | G |  | Frameshift in BPI_I1126, hypothetical protein pseudogene (orth: BMI_I1090). |
| 1070587 | -> | 1091311 | 1091311 |  | G | Frameshift in BPI_I1137, TetR family transcriptional regulator, pseudogene in B. microti (orth: BMI_I1102) |
| 1110783 | 1110783 | -> | 1131506 | T |  | Frameshift in BPI_I1174, branched-chain alpha-keto acid dehydrogenase subunit E2. pseudogene BMI_I1139 in B. microti. |
| 1148386 | 1148386 | -> | 1169108 | G |  | Frameshift in BPI_I1209, hypothetical protein pseudogene (orth: BMI_I1174) |
| 1149326 | -> | 1170049 | 1170054 |  | TGCAGA | In frame change in BPI_I1210, ATP/GTP-binding motif-containing protein (orth: BMI_I1175) |
| 1152625 | 1152625 | -> | 1173352 | T |  | Intergenic |
| 1158603 | 1158632 | -> | 1179328 | AGAGAAGCAGCCGTAACGGCCTTGACCGCA |  | In frame change in BPI_1223, L-serine dehydratase, iron-sulfur-dependent, single chain form (orth: BMI_I1186) |
| 1161518 | 1161518 | -> | 1182213 | G |  | Frameshift in BPI_I1224, hypothetical protein pseudogene (orth: BMI_I1189) |
| 1164292 | -> | 1184988 | 1185831 |  | GGGCGTGTCTGCATTCAACGCAACCAGATCATAGCGCATGCGAGATGGACGAAGCCCATGAATGCGGTCAATGTTTTCTCGCATCGCAGCGCAATACGACGATAGCGTTTCAACTTGTTAAAAAAGCATTCAATCTGATGGCGTTCCTTGTACAGCCTCCAGTCGATTGTTGGGACACTGGAACGTGTTGGATTGGCCTTGATCTGAGCCGTTGCCTTGAGATTGCTGGCAATGAAGGCCCTTAAGTGATCGGCATCATAGGCCGCATCAGCAATGACATGCCCCACACCCTCTAAGCCGGATAGAAGGCTTGAAGCTTGCGGACAGTCACCATAATGGCCGGGTGTTGGCTTTATTCGCAGCGGTAGGCCGATAGCATCGACAACAGCATGCAGCTTGGTCGTCAATCCACCGCGCGAGCGACCGATGCAGGCAGCTTCAGCCCCCCTTTTGCGCCCGCCGCATCTGCGTGGACTTTCGATATGGTGCTATCAATGAGGACATATTCAAAGTCCGGCGTATCAGCCAGGGCATGGAAAAGCCTTTCCCATACACCGGCGTGCGACCAGCGCCGAAAGCGGGCATGAACCGCTGTCCATTTGCCGAAGGTCGCAGGCAGATCGCGCCAGTGCGCTGCATTGGCAGCCATCCACAAGATGGCGTCGACAAATAATCGGTTATCGACGCCACTGCGGCCGGGCGTACCAACTCGCCCCGGAAGATATGCTTCGATCCGGTTCCATTGCTCATCTGTAAGGCTTCGTCTGCTCACGGCTGTTCTCCTTCAACAACCTTGAATCAGAATTTCGTACAAAAGGGAATCCTTGAATGCAGACAAGCCCTA | IS711 family transposase |
| 1164404 | -> | 1185945 | 1185945 |  | G | Intergenic |
| 1177306 | 1177328 | -> | 1198846 | TCGCTTGCCGACAAGCGCCGCCA |  | Intergenic |
| 1212166 | 1212183 | -> | 1233683 | CGACGAAGAGGAAGAGGA |  | In frame change in BPI_I1288, hypothetical protein (orth: BMI_I1251) |
| 1231687 | -> | 1253188 | 1254031 |  | GGCGTGTCTGCATTCAACGCAACCAGATCATAGCGCATGCGAGATGGACGAAGCCCATGAATGCGGTCAATGTTTTCTCGCATCGCAGCGCAATACGACGATAGCGTTTCAACTTGTTAAAAAAGCATTCAATCTGATGGCGTTCCTTGTACAGCCTCCAGTCGATTGTTGGGACACTGGAACGTGTTGGATTGGCCTTGATCTGAGCCGTTGCCTTGAGATTGCTGGCAATGAAGGCCCTTAAGTGATCGGCATCATAGGCCGCATCAGCAATGACATGCCCCACACCCTCTAAGCCGGATAGAAGGCTTGAAGCTTGCGGACAGTCACCATAATGGCCGGGTGTTGGCTTTATTCGCAGCGGTAGGCCGATAGCATCGACAACAGCATGCAGCTTGGTCGTCAATCCACCGCGCGAGCGACCGATGCAGGCAGCTTCAGCCCCCCTTTTGCGCCCGCCGCATCTGCGTGGACTTTCGATATGGTGCTATCAATGAGGACATATTCAAAGTCCGGCGTATCAGCCAGGGCATGGAAAAGCCTTTCCCATACACCGGCGTGCGACCAGCGCCGAAAGCGGGCATGAACCGCTGTCCATTTGCCGAAGGTCGCAGGCAGATCGCGCCAGTGCGCTGCATTGGCAGCCATCCACAAGATGGCGTCGACAAATAATCGGTTATCGACGCCACTGCGGCCGGGCGTACCAACTCGCCCCGGAAGATATGCTTCGATCCGGTTCCATTGCTCATCTGTAAGGCTTCGTCTGCTCACGGCTGTTCTCCTTTAACAACCTTGAATCAGAATTTCGTACAAAAGGGAATCCTTGAATGCAGACAAGCCCTAG | IS711 family transposase |
| 1240580 | 1240610 | -> | 1262923 | GATGCCATGACTACCTCGATATAATTTGAAC |  | Intergenic |
| 1240790 | -> | 1263104 | 1263947 |  | GGCGTGTCTGCATTCAACGCAACCAGATCATAGCGCATGCGAGATGGACGAAGCCCATGAATGCGGTCAATGTTTTCTCGCATCGCAGCGCAATACGACGATAGCGTTTCAACTTGTTAAAAAAGCATTCAATCTGATGGCGTTCCTTGTACAGCCTCCAGTCGATTGTTGGGACACTGGAACGTGTTGGATTGGCCTTGATCTGAGCCGTTGCCTTGAGATTGCTGGCAATGAAGGCCCTTAAGTGATCGGCATCATAGGCCGCATCAGCAATGACATGCCCCACACCCTCTAAGCCGGATAGAAGGCTTGAAGCTTGCGGACAGTCACCATAATGGCCGGGTGTTGGCTTTATTCGCAGCGGTAGGCCGATAGCATCGACAACAGCATGCAGCTTGGTCGTCAATCCACCGCGCGAGCGACCGATGCAGGCAGCTTCAGCCCCCCTTTTGCGCCCGCCGCATCTGCGTGGACTTTCGATATGGTGCTATCAATGAGGACATATTCAAAGTCCGGCGTATCAGCCAGGGCATGGAAAAGCCTTTCCCATACACCGGCGTGCGACCAGCGCCGAAAGCGGGCATGAACCGCTGTCCATTTGCCGAAGGTCGCAGGCAGATCGCGCCAGTGCGCTGCATTGGCAGCCATCCACAAGATGGCGTCGACAAATAATCGGTTATCGACGCCACTGCGGCCGGGCGTACCAACTCGCCCCGGAAGATATGCTTCGATCCGGTTCCATTGCTCATCTGTAAGGCTTCGTCTGCTCACGGCTGTTCTCCTTTAACAACCTTGAATCAGAATTTCGTACAAAAGGGAATCCTTGAATGCAGACAAGCCCTAG | IS711 family transposase |
| 1246069 | 1246080 | -> | 1269225 | CACTGGCACCGG |  | In frame change in BPI_I1329, putative peptidase M15A (orth: BMI_I1288) |
| 1248347 | -> | 1271493 | 1271512 |  | CATTTCCGGCAAATCTAGAG | Intergenic |
| 1251780 | 1252622 | -> | 1274944 | CTGGAAGATATGCTTTAGGGCCTGTCTGCATTTAACGTAACCAGATCATAGCGCATGCGAGATGGACGAAACCCATGAATGCGGTCAATGTTTTCTCGCATCGCAGCGCAATACGACGATAGCGTTTCAACTTGTTAAAAAAGCATTCAATCATCGATGGCGTTCCTTGGACAGCCTCCAGTCGATTGTTGGGACACTGGAACGTGTTGGATTGGCCTTGGATCTGAGCCGTTGCCATGAGATCGCTGGCAATGAAGGCCCTTAAGTGATCGGCATCATGGGCCGCGTCAGCAATGACATGTCCCACACCCTTTAAGCCGGATAGAAGGCTTGAAGCTTGCGGACAGTCACCATAATGGCCGGGTGTTGGCTTTATTCGCAGCGGTAGGCCGATAGCATCGACAACAGCATGCAGCTTGGTCGTCAATCCACCGCGCGAGCGACCGATGCAGGCAGCTTCAGCCCCCCTTTTGCGCCCGCCGCATCTGCGTGGACTTTCGATATGGTGCTATCAATGAGGACATATTCAAAGTCCGGCGTATCAGCCAGGGCATGGAAAAGCCTTTCCCATACACCGGCGTGCGACCAGCGCCGAAAGCGGGCATGAACCGCTGTCCATTTGCCGAAGGTCGCAGGCAGATCGCGCCAGTGCGCTGCATTGGCAGCCATCCACAAGATGGCGTCGACAAATAATCGGTTATCGACGCCACTGCGGCCGGGCGTACCAACTCGCCCCGGAAGATATGCTTCGATCCGGTTCCATTGCTCATCTGTAAGGCTTCGTCTGCTCACGGCTGTTCTCCTTCAACAACCTTGAATCAGAATTTCATGCAAAAGGGAATC |  | IS711 family transposase |
| 1252629 | 1252632 | -> | 1274950 | TGCA |  | Intergenic |
| 1279773 | -> | 1302092 | 1302935 |  | GGCTTGTCTGCATTCAAGGATTCCCTTTTGTACGAAATTCTGATTCAAGGTTGTTAAAGGAGAACAGCCGTGAGCAGACGAAGCCTTACAGATGAGCAATGGAACCGGATCGAAGCATATCTTCCGGGGCGAGTTGGTACGCCCGGCCGCAGTGGCGTCGATAACCGATTATTTGTCGACGCCATCTTGTGGATGGCTGCCAATGCAGCGCACTGGCGCGATCTGCCTGCGACCTTCGGCAAATGGACAGCGGTTCATGCCCGCTTTCGGCGCTGGTCGCACGCCGGTGTATGGGAAAGGCTTTTCCATGCCCTGGCTGATACGCCGGACTTTGAATATGTCCTCATTGATAGCACCATATCGAAAGTCCACGCAGATGCGGCGGGCGCAAAAGGGGGGCTGAAGCTGCCTGCATCGGTCGCTCGCGCGGTGGATTGACGACCAAGCTGCATGCTGTTGTCGATGCTATCGGCCTACCGCTGCGAATAAAGCCAACACCCGGCCATTATGGTGACTGTCCGCAAGCTTCAAGCCTTCTATCCGGCTTAGAGGGTGTGGGGCATGTCATTGCTGATGCGGCCTATGATGCCGATCACTTAAGGGCCTTCATTGCCAGCAATCTCAAGGCAACGGCTCAGATCAAGGCCAATCCAACACGTTCCAGTGTCCCAACAATCGACTGGAGGCTGTACAAGGAACGCCATCAGATTGAATGCTTTTTTAACAAGTTGAAACGCTATCGTCGTATTGCGCTGCGATGCGAGAAAACATTGACCGCATTCATGGGCTTCGTCCATCTCGCATGCGCTATGATCTGGTTGCGTTGAATGCAGACACGCCCTAG | IS711 family transposase |
| 1298727 | 1298900 | -> | 1321888 | GTTGCGGACTGGCCCCGTGTTGCGGGCCTGCAAGGCTGCGTCAGCGTTCTGCATAGCGAAGCCGCCTTGCCTGGATGAGATTGGTCACGAGAAGCATGAAAAAAGCCATCACCAGCATCACGAAGGCCAGTGCCGCCGCCGCCGGATAATCATATTCATCAAGCCGGATGAAAA |  | In frame change in BPI_I1382, sulfate transport system permease protein CysT (orth: BMI_I1341) |
| 1301015 | -> | 1324004 | 1324004 |  | T | Intergenic |
| 1308590 | 1308629 | -> | 1331578 | CATAGACCCAATGGAAAGATCGAACTCGCCGCCGATCATC |  | Frameshift in BPI_I1392, sugar ABC transporter, permease protein pseudogene (orth: BMI_I1352). |
| 1318218 | 1318226 | -> | 1341166 | AGCATGGCC |  | In frame change in BPI_I1401, ABC transporter, ATP binding/permease protein, (orth: BMI_I1361) |
| 1322561 | 1322561 | -> | 1345500 | A |  | Frameshift in BPI_I1404, hypothetical protein pseudogene (orth: BMI_I1364) |
| 1323567 | 1323714 | -> | 1346505 | CACGGACCACCCTATCGCCCTTTCATAAATTAAACTTCCCAATCTTGACGTGAGCAAAGATAGCCCTAGCTCAACCTGTCTGACGCGGTTTACTTTCCGGTTTTAAGTACCGGAAACGGCAATGTTATGATGGGTGCCTCGATTGGAG |  | Intergenic |
| 1334399 | -> | 1357191 | 1357191 |  | G | Frameshift in BPI_I1419, CrcB family protein (orth: BMI_I1379) |
| 1342678 | 1342765 | -> | 1365469 | TCCGGCGGCGCCGGGCAAGCAAAAGAAAAGTGCATCGAACTAATCAGTTGCGGGCTTTATCGACCAGTTTGTTCTTTTCGATCACCCG |  | No change |
| 1343907 | 1343907 | -> | 1366610 | A |  | Frameshift in BPI_I1431, transcriptional regulator, TetR family pseudogene (orth: BMI_I1391) |
| 1344723 | 1344723 | -> | 1367425 | T |  | Frameshift in BPI_I1432 (orth: BMI_I1392), the resulting orf is longer in B. microti than in B. pinnipedialis. Hypothetical proteins. |
| 1359685 | -> | 1382388 | 1382390 |  | GCC | In frame change in BPI_I1448, HflK protein (orth: BMI_I1408) |
| 1365856 | 1365856 | -> | 1388560 | C |  | Slightly change the end of BPI_I1459, hypothetical protein, longuer in B. microti (orth: BMI_I1419). |
| 1370785 | 1370785 | -> | 1393488 | C |  | Frameshift in BPI_I1462, hypothetical protein pseudogene (orth: BMI_I1422) |
| 1387612 | 1387619 | -> | 1410314 | AATCGTTG |  | Intergenic |
| 1406973 | 1406988 | -> | 1429667 | AGGGCAGTAGGGCAGT |  | Intergenic |
| 1407059 | 1407065 | -> | 1429736 | TATTCCC |  | Intergenic |
| 1407107 | 1407113 | -> | 1429776 | TGCCTTA |  | Intergenic |
| 1415400 | 1415492 | -> | 1438062 | GCCTCTCCATCCCCGCTCCGGCAGAAACCTTCCTGAAGATGCTGGGCGGTGCGGCCTCGCCTTGCGCACTGGTCACGCTTGGACTCTTTCTCG |  | In frame change in BPI_I1501, auxin efflux carrier (orth: BMI_I1461) |
| 1434537 | 1434537 | -> | 1457106 | T |  | Difference in the beginning of BPI_I1522, hypothetical protein (orth: BMI_I1483) |
| 1436888 | 1437731 | -> | 1459455 | GGCCTGTCTGCATTCAACGCAACCAGATCATAGCGCATGCGAGATGGACGAAACCCATGAATGCGGTCAATGTTTTCTCGCATCGCAGCGCAATGCGACGATAGCGTTTCAACTTGTTAAAAAAGCATTCAATCTGATGGCGTTCCTTGTACAGCCTCCAGTCGATTGTTGGGGCACTGGAACGTGTTGGATTGGCCTTGGATCTGAGCCGTTGCCTTGAGATCGCTGGCAATGAAGGCCCTTAAGTGATCGGCATCATGGGCCGCGTCAGCAATGACATGTCCCACACCCTTTAAGCCGGATAGAAGGCTTGAAGCTTGCGGACAGTCACCATAATGGCCGGGTGTTGGCTTTATTCGCAGCGGTAGGCCGATAGCATCGACAACAGCATGCAGCTTGGTCGTCAATCCACCGCGCGAGCGACCGATGCAGGCAGCTTCAGCCCCCCTTTTGCGCCCGCCGCATCTGCGTGGACTTTCGATATGGTGCTATCAATGAGGACATATTCAAAGTCCGGCGTATCAGCCAGGGCATGGAAAAGCCTTTCCCATACACCGGCGTGCGACCAGCGCCGAAAGCGGGCATGAACCGCTGTCCATTTGCCGAAGGTCGCAGGCAGATCGCGCCAGTGCGCTGCATTGGCAGCCATCCACAAGATGGCGTCGACAAATAATCGGTTATCGACGCCACTGCGGCCGGGCGTACCAACTCGCCCCGGAAGATATGCTTCGATCCGGTTCCATTGCTCATCTGTAAGGCTTCGTCTGCTCACGGCTGTTCTCCTTCAACAACCTTGAATCAGAATTTCATGCAAAAGGGAATCCTTGTATGCAGACAGGCCCTA |  | IS711 family transposase |
| 1438771 | -> | 1460496 | 1461339 |  | GGCGTGTCTGCATTCAACGCAACCAGATCATAGCGCATGCGAGATGGACGAAGCCCATGAATGCGGTCAATGTTTTCTCGCATCGCAGCGCAATACGACGATAGCGTTTCAACTTGTTAAAAAAGCATTCAATCTGATGGCGTTCCTTGTACAGCCTCCAGTCGATTGTTGGGACACTGGAACGTGTTGGATTGGCCTTGATCTGAGCCGTTGCCTTGAGATTGCTGGCAATGAAGGCCCTTAAGTGATCGGCATCATAGGCCGCATCAGCAATGACATGCCCCACACCCTCTAAGCCGGATAGAAGGCTTGAAGCTTGCGGACAGTCACCATAATGGCCGGGTGTTGGCTTTATTCGCAGCGGTAGGCCGATAGCATCGACAACAGCATGCAGCTTGGTCGTCAATCCACCGCGCGAGCGACCGATGCAGGCAGCTTCAGCCCCCCTTTTGCGCCCGCCGCATCTGCGTGGACTTTCGATATGGTGCTATCAATGAGGACATATTCAAAGTCCGGCGTATCAGCCAGGGCATGGAAAAGCCTTTCCCATACACCGGCGTGCGACCAGCGCCGAAAGCGGGCATGAACCGCTGTCCATTTGCCGAAGGTCGCAGGCAGATCGCGCCAGTGCGCTGCATTGGCAGCCATCCACAAGATGGCGTCGACAAATAATCGGTTATCGACGCCACTGCGGCCGGGCGTACCAACTCGCCCCGGAAGATATGCTTCGATCCGGTTCCATTGCTCATCTGTAAGGCTTCGTCTGCTCACGGCTGTTCTCCTTTAACAACCTTGAATCAGAATTTCGTACAAAAGGGAATCCTTGAATGCAGACAAGCCCTAG | IS711 family transposase |
| 1458682 | 1458682 | -> | 1481249 | G |  | Intergenic |
| 1483321 | -> | 1505889 | 1505950 |  | TCCTGAAAAGTGTGAAACGGTTTTCGGAAAAGATGCGCGTCAAAGCAAAGAATTAGAGCCCA | Intergenic |
| 1509485 | 1509485 | -> | 1532113 | T |  | Frameshift in BPI_I1606, aspartyl/asparaginyl beta-hydroxylase pseudogene (orh: BMI_I1566) |
| 1512681 | -> | 1535310 | 1535310 |  | G | Intergenic |
| 1534969 | 1534970 | -> | 1557597 | AC |  | Slight change in the beginning of gene BPI_I1628, hypothetical protein (orth: BMI_I1588) |
| 1541241 | -> | 1563869 | 1563869 |  | G | Frameshift in BPI_I1637, dipeptide transport system permease protein DppC, pseudogene BMI_I1597 in B. microti. |
| 1544274 | 1544274 | -> | 1566901 | C |  | Intergenic |
| 1545072 | 1545073 | -> | 1567697 | TG |  | Frameshift in BPI_I1641, hypothetical protein, pseudogene BMI_I1601 in B. microti. |
| 1571899 | 1572148 | -> | 1594522 | CCACGAAAAATGATGGCGCTTTCAACAATTGCCGTCGCCGCCATGTTTCTTCTCTTTGTGTGCCTTCGGCAGCACGGGAACGGCGACGGAAACGGGGGCGATCTCTTGATCCCGGTCATCGTCACACACGAAAAATGATGGCGCTCTCAACAATTGCCGTCGCCGCCATGTCCCTTCTCTTTGTGTGCCTTCGGCAGCACGGGAACGGCGATGGAAACGGGGGCGATCTCTTGATCCCGGTCATCGTCAC |  | Region orthologous to BMI_I1632 is not present in B. pinnipedialis. |
| 1573409 | 1573409 | -> | 1595783 | G |  | Frameshift in BPI_I1674, 7-alpha-hydroxysteroid dehydrogenase pseudogene (orth: BMI_I1635). |
| 1572596 | -> | 1594971 | 1594971 |  | A | Intergenic |
| 1595933 | -> | 1618308 | 1618321 |  | GTGCTGTTCTCGCT | In 23S ribosomal rna |
| 1595943 | -> | 1618332 | 1618339 |  | ACGCATTC | In 23S ribosomal rna |
| 1595970 | 1595983 | -> | 1618365 | CAATAAGGCAATAT |  | In 23S ribosomal rna |
| 1595996 | 1595997 | -> | 1618377 | CT |  | In 23S ribosomal rna |
| 1596017 | -> | 1618398 | 1618398 |  | G | In 23S ribosomal rna |
| 1596030 | -> | 1618412 | 1618428 |  | CCATCCAGCGTTGCTCC | In 23S ribosomal rna |
| 1599646 | 1599646 | -> | 1622044 | T |  | Frameshift in BPI_I1700, transcriptional regulator, IclR family pseudogene (orth: BMI_I1661) |
| 1599416 | -> | 1621815 | 1621815 |  | T | as above |
| 1607475 | 1607475 | -> | 1629872 | G |  | Frameshift in BPI_I1708, malate synthase pseudogene (orth: BMI_I1669) |
| 1608538 | 1608538 | -> | 1630934 | T |  | Intergenic |
| 1614867 | -> | 1637264 | 1638107 |  | TTGTCTGCATTCAAGGATTCCCTTTTGTACGAAATTCTGATTCAAGGTTGTTAAAGGAGAACAGCCGTGAGCAGACGAAGCCTTACAGATGAGCAATGGAACCGGATCGAAGCATATCTTCCGGGGCGAGTTGGTACGCCCGGCCGCAGTGGCGTCGATAACCGATTATTTGTCGACGCCATCTTGTGGATGGCTGCCAATGCAGCGCACTGGCGCGATCTGCCTGCGACCTTCGGCAAATGGACAGCGGTTCATGCCCGCTTTCGGCGCTGGTCGCACGCCGGTGTATGGGAAAGGCTTTTCCATGCCCTGGCTGATACGCCGGACTTTGAATATGTCCTCATTGATAGCACCATATCGAAAGTCCACGCAGATGCGGCGGGCGCAAAAGGGGGGCTGAAGCTGCCTGCATCGGTCGCTCGCGCGGTGGATTGACGACCAAGCTGCATGCTGTTGTCGATGCTATCGGCCTACCGCTGCGAATAAAGCCAACACCCGGCCATTATGGTGACTGTCCGCAAGCTTCAAGCCTTCTATCCGGCTTAGAGGGTGTGGGGCATGTCATTGCTGATGCGGCCTATGATGCCGATCACTTAAGGGCCTTCATTGCCAGCAATCTCAAGGCAACGGCTCAGATCAAGGCCAATCCAACACGTTCCAGTGTCCCAACAATCGACTGGAGGCTGTACAAGGAACGCCATCAGATTGAATGCTTTTTTAACAAGTTGAAACGCTATCGTCGTATTGCGCTGCGATGCGAGAAAACATTGACCGCATTCATGGGCTTCGTCCATCTCGCATGCGCTATGATCTGGTTGCGTTGAATGCAGACACGCCCTAGGGC | IS711 family transposase |
| 1625415 | 1625415 | -> | 1648654 | T |  | Frameshift in BPI_I1735, efflux transporter, RND family pseudogene, also pseudogene BMI_I1694 in B. microti. |
| 1656749 | -> | 1679988 | 1680000 |  | ACGAAGACCGGCA | Frameshift in BPI_I1767, OpgC protein, pseudogene BMI_I1726 in B. microti. |
| 1690383 | 1690398 | -> | 1713633 | GAAGCAGATTCCGCCG |  | Frameshift in BPI_I1808, pyruvate kinase pseudogene (orth: BMI_I1767) |
| 1698642 | 1698670 | -> | 1721876 | GCGCTGCCGATTGCAGGCGTCGTCGTTTC |  | Frameshift in BPI_I1818, thiamin ABC transporter, membrane component and ATP-binding component, merge genes ThiP and ThiQ in B. microti (BMI_I1777 and BMI_I1778) |
| 1704900 | 1704900 | -> | 1728105 | T |  | Intergenic |
| 1778877 | 1778952 | -> | 1802081 | TTTCTGCGTCGCATCATTTCGCGCATCGATGCCGTGCGAAGGGTCGCCTTTTCGTCGATCATCTCCGTTTTCTGCC |  | Permature stop at the end of BPI_I1895, hypothetical protein (orth: BMI_I1855) |
| 1785633 | 1786010 | -> | 1808761 | CAAGAGCGGAAATCGCAGCACTATTCTGTCTGATAGCAGCCCTATTGCTAGCAACACCTGCTGCGTTAGCATTGATGCGACCGCGGTTTTGGCTGATGGCAGCCCTATTGTCGGCAACACCTTTGGCATTGGTATCAATCCGGCCACTGTTTCGACCAATGGCAGCCCTATTGTCAGCAACGCCCTTGGCATTGGTGTCAATCCGGCCGCTGTTCCGGCCAATGGCAGCCCGGTTGTCGGCAACACCTTTGGCATTGGTGTCGATCCGACCGCTGTTCCGGCCAATGGCAGTCTTGTTGTCGGCAACGCCCTTGGCATTGGCGTCGATCCGGACACTATTCTGGCCAATGGCAGTCTTGTTGTCGGCAACGCCCTTGG |  | Large deletion in BPI_I1902, hypothetical protein (orth: BMI_I1862) |
| 1786827 | 1786827 | -> | 1809577 | A |  | Intergenic |
| 1795275 | -> | 1818026 | 1818026 |  | T | Frameshift in BPI_I1913, hypothetical protein, pseudogene BMI_I1873 in B. microti. |
| 1800625 | -> | 1823377 | 1823390 |  | GTGCTGTTCTCGCT | Change in 23S ribosomal RNA |
| 1800635 | -> | 1823401 | 1823408 |  | ACGCATTC | Change in 23S ribosomal RNA |
| 1800662 | 1800675 | -> | 1823434 | CAATAAGGCAATAT |  | Change in 23S ribosomal RNA |
| 1800688 | 1800689 | -> | 1823446 | CT |  | Change in 23S ribosomal RNA |
| 1800709 | -> | 1823467 | 1823467 |  | G | Change in 23S ribosomal RNA |
| 1800722 | -> | 1823481 | 1823497 |  | CCATCCAGCGTTGCTCC | Change in 23S ribosomal RNA |
| 1807684 | 1807684 | -> | 1830458 | T |  | Change in 23S ribosomal RNA |
| 1823468 | 1824311 | -> | 1846241 | GGCTTGTCTGCATTCAAGGATTCCCTTTTGCATGAAATTCTGATTCAAGGTTGTTGAAGGAGAACAGCCGTGAGCAGACGAAGCCTTACAGATGAGCAATGGAACCGGATCGAAGCATATCTTCCGGGGCGAGTTGGTACGCCCGGCCGCAGTGGCGTCGATAACCGATTATTTGTCGACGCCATCTTGTGGATGGCTGCCAATGCAGCGCACTGGCGCGATCTGCCTGCGACCTTCGGCAAATGGACAGCGGTTCATGCCCGCTTTCGGCGCTGGTCGCACGCCGGTGTATGGGAAAGGCTTTTCCATGCCCTGGCTGATACGCCGGACTTTGAATATGTCCTCATTGATAGCACCATATCGAAAGTCCACGCAGATGCGGCGGGCGCAAAAGGGGGGCTGAAGCTGCCTGCATCGGTCGCTCGCGCGGTGGATTGACGACCAAGCTGCATGCTGTTGTCGATGCTATCGGCCTACCGCTGCGAATAAAGCCAACACCCGGCCATTATGGTGACTGTCCGCAAGCTTCAAGCCTTCTATCCGGCTTAAAGGGTGTGGGACATGTCATTGCTGACGCGGCCTATGATGCCGATCACTTAAGGGCCTTCATTGCCAGCAATCTCAAGGCAACGGCTCAGATCAAGGTCAATCCAACACGTTCCAGTGTCCCAACAATCGACTGGAGGCTGTACAAGGAACGCCATCAGATTGAATGCTTTTTTAACAAGTTGAAACGCTATCGTCGTATTGCGCTGCGATGCGAGAAAACATTGACCGCATTCATGGGTTTCGTCCATCTCGCATGCGCTATGATCTGGTTACGTTAAATGCAGACACGCCCTAG |  | IS711 family transposase |
| 1849154 | 1849172 | -> | 1871083 | TCATGGGCGTCGCGCAAAT |  | Frameshift makes BPI_I1964, GCN5-related N-acetyltransferase, different in the beginning from its B. microti counterpart (BMI_I1927). |
| 1851550 | 1851552 | -> | 1873460 | AAA |  | Intergenic |
| 1870848 | 1870876 | -> | 1892755 | GCGCCTACATTTTGAATTTCTCTCAGGCT |  | Slightly changes the end of BPI_I1987, hypothetical protein, with respect to its ortholog BMI_I1950. |
| 1882789 | 1882797 | -> | 1904667 | GCTGCAAGG |  | In frame change in BPI_I2002, peptidyl-prolyl cis-trans isomerase (orth: BMI_I1965). |
| 1905284 | 1905367 | -> | 1927153 | GGCTGCCCAGAAGCAGCTTGTTGACGCCGCGCCGCCCGTGCGAAGCAATCACAATCAGATCGCAGTCCTTCGCTTTCGCCGCCT |  | In frame change in BPI_I2007, preprotein translocase subunit SecA (orth: BMI_I1967) |
| 1922366 | 1922380 | -> | 1944151 | AAGGTCAGAGTCAGG |  | In frame change in BPI_I2044, hypothetical protein (orth: BMI_I2007) |
| 1926279 | -> | 1948051 | 1948051 |  | C | Frameshift prolongating BPI_I2048, prephenate dehydrogenase TyrC (orth: BMI_I2011). Change in the 10 last amino-acids. |
| 1931406 | 1931406 | -> | 1953177 | A |  | Intergenic |
| 1939535 | 1939537 | -> | 1961305 | TGG |  | In frame change in BPI_I2068, intimin/invasin family protein (orth: BMI_I2031) |
| 1944590 | 1944590 | -> | 1966357 | G |  | Frameshift in BPI_I2072, outer membrane autotransporter pseudogene (orth: BMI_I2035) |
| 1969605 | 1969986 | -> | 1991370 | ACCAGCAGATGGTCAATGCTCACCTCGCCGAACTCGTTGATAATGACTGCTGTATCGCTGAGCGCCGGATCTTTCAAAAGCCGGTTGAGCAGCGTCGTCTTGCCGGAGCCAAGAAAGCCGGTCAGCACGGAAACGGGAATGGGATCAGGCATATCAGTTGATGGCCGTTGTCTTTACGCCTGGCGCGCGCTGCGGACGCGGCATTGGCACCGGAACCCGCGAAATGCCGGGCTGGCCGGGCCGGTTGATCGAACCCGGTTCGGAAACCAGCCTCACCTGCACGGCGATCGGATCATGGTCCATGGCGTGAATATAGGGTGAATTGATCTTCAGCCGGCCTTCTACCTCGCGCCCGTCCCAGCGGTCGGCCATGGCCTGCTCG |  | In BPI_I2093, cobalamin synthesis protein P47K pseudogene. |
| 1972250 | 1972250 | -> | 1993633 | C |  | Frameshift in BPI_I2094, amidohydrolase which is a pseudogene in B. microti (BMI_I2058). |
| 1984424 | -> | 2005808 | 2005808 |  | T | Intergenic |
| 1988486 | 1988486 | -> | 2009870 | A |  | Intergenic |
| 1986164 | -> | 2007549 | 2007549 |  | C | Frameshift in BPI_I2109, bacterial luciferase family protein pseudogene (orth: BMI_I2073) |
| 2015219 | -> | 2036604 | 2036604 |  | G | Intergenic |
| 2021775 | -> | 2043161 | 2043170 |  | GAAATTTTTT | Intergenic |
| 2052733 | -> | 2074129 | 2074141 |  | TGGCGATAAATTT | Intergenic |
| 2053635 | 2053714 | -> | 2075042 | AAGAGGCGTGACGTGCCGTAGGCACGGCAGGGATATAATCAAGAGGCGTGGAGTGCCGTAGGCGCGACAGGGATTTAACC |  | Intergenic |
| 2066992 | 2066992 | -> | 2088319 | G |  | Frameshift in BPI_I2190, Mg chelatase-related protein, which is pseudogene BMI_I2154 in B. microti. |
| 2077267 | 2077267 | -> | 2098593 | G |  | Intergenic |
| 2096233 | -> | 2117560 | 2117610 |  | CTGGCCGCCCGCAAGGTGGTCGCCCGCAGCCGGCCGGAAGGCCGCAGCAGG | In frame change in BPI_I2222, translation initiation factor IF-2 (orth: BMI_I2186) |
| 2110433 | 2110537 | -> | 2131809 | CATATTGGGCGGGATTTTCCACCGGAGCGCCGTGGTTTGAATTGGGCAGGATAATCAATTCCGCGCCCGGAATCTGCTGCGCGATGCATTCGGTATGCGCACGGG |  | In frame change in BPI_I2235, putative hydrolase (orth: BMI_I2199). |
| 2113767 | -> | 2135040 | 2135059 |  | AGCACCAGCACACGCCCGGC | Frameshift in BPI_I2239, cytosol aminopeptidase family protein, changes the start of the encoded protein. |
