# Supplementary material for: The genome sequence of Brucella pinnipedialis B2/94 sheds light on the evolutionary history of the genus Brucella
Source: BMC Evol Biol. 2011 Jul 11;11:200. doi: 10.1186/1471-2148-11-200 (PMC3146883; doi:10.1186/1471-2148-11-200)
Supplement: Additional file 2 — Indels in B. pinnipedialis B2/94 and B. microti small chromosome alignment. List of insertions and deletions (indels) in the complete genome alignment of B. pinnipedialis B2/94 and B. microti CCM 4915, small chromosome. [file 1471-2148-11-200-S2.DOC]

| **Indels in small chromosome alignment of *B. pinnipedialis* and *B. microti*** | | | | | | |
| --- | --- | --- | --- | --- | --- | --- |
| **Coordinates *B. microti*** | | **Coordinates *B. pinnipedialis*** | | **Indel *B. microti*** | **Indel *B. pinnipedialis*** | **Comment** |
| 9091 | -> | 9092 | 9092 |  | A | Intergenic |
| 15077 | -> | 15079 | 15079 |  | C | Intergenic |
| 20868 | 20868 | -> | 20870 | A |  | Frameshift in BPI_II24, branched chain amino acid ABC transporter, periplasmic amino acid-binding protein pseudogene. Ortholog is BMI_II24 in B. microti. |
| 19730 | -> | 19733 | 19733 |  | G | Frameshift in BPI_II22, extracytoplasmic function alternative sigma factor, changes end of gene (orth: BMI_II22). |
| 54857 | 54857 | -> | 54858 | T |  | Frameshift in BPI_II59, hypothetical protein, changes the end of the gene. Other Brucella are most similar to B. microti (orth: BMI_II59). |
| 58820 | 58828 | -> | 58822 | CTGGTGGTG |  | In frame change in BPI_II62, type IV secretion system protein VirB10 (orth: BMI_II62) |
| 56807 | -> | 56809 | 56810 |  | GC | Intergenic |
| 66137 | 66137 | -> | 66130 | T |  | Intergenic |
| 73072 | 73111 | -> | 73064 | GGGAGTAAGGGAGTAAGGGAGTAAGGGAGTAAGGGAGTAA |  | Intergenic |
| 73208 | -> | 73162 | 73177 |  | TTCCCCTATTCCCCTA | small duplication in B. pinnipedialis, do not change the end of the gene BPI_II76 (orth: BMI_II76) |
| 75896 | 75896 | -> | 75864 | A |  | Intergenic |
| 84694 | -> | 84663 | 84739 |  | ACGGCGCGCCCGGCGCGGGCAACCGCGCCCACGCGGCAGGCCCCGGCAGCCGTGCCGGCGGCGCCTGTGGAACAGCC | Fragment of BPI_II85, deleted from B. microti (orth: BMI_II85), hypothetical protein. |
| 90836 | 90837 | -> | 90880 | CG |  | Frameshift in BPI_II92, molybdenum ABC transporter, ATP-binding protein pseudogene. B.microti ortholog BMI_II92 is intact. |
| 92482 | 92483 | -> | 92524 | TA |  | Frameshift in BPI_II95, pseudogene corresponding to Meso_1225 in Mesorhizobium sp. BNC1, hypothetical protein and alos pseudogene in other Brucella (orth: BMI_II95). |
| 97039 | -> | 97080 | 97080 |  | A | Intergenic |
| 116968 | -> | 117010 | 117010 |  | A | Frameshift in BPI_II117, HlyD family secretion protein, pseudogene in B. microti (BMI_II117). |
| 118148 | 118148 | -> | 118189 | T |  | Intergenic |
| 118232 | 118232 | -> | 118272 | A |  | Intergenic |
| 121135 | -> | 121176 | 121190 |  | ATCGTCGCGGTTGAG | In frame change in BPI_II122, flagellar motor switch protein G (orth: BMI_II122). |
| 140057 | 140125 | -> | 140111 | ATCGATTGCCAAAGCCTGTGTCACAGCCCCGGATGTTTCTCCGCCTGCAACAATGAAGCGCGTAAACCC |  | In frame change in BPI_II146, hypothetical protein pseudogene (orth: BMI_II146) |
| 140967 | -> | 140954 | 140954 |  | T | Frameshift in BPI_II146, hypothetical protein pseudogene (orth: BMI_II146). |
| 154401 | -> | 154389 | 155232 |  | GGCTTGTCTGCATTCAAGGATTCCCTTTTGTACGAAATTCTGATTCAAGGTTGTTAAAGGAGAACAGCCGTGAGCAGACGAAGCCTTACAGATGAGCAATGGAACCGGATCGAAGCATATCTTCCGGGGCGAGTTGGTACGCCCGGCCGCAGTGGCGTCGATAACCGATTATTTGTCGACGCCATCTTGTGGATGGCTGCCAATGCAGCGCACTGGCGCGATCTGCCTGCGACCTTCGGCAAATGGACAGCGGTTCATGCCCGCTTTCGGCGCTGGTCGCACGCCGGTGTATGGGAAAGGCTTTTCCATGCCCTGGCTGATACGCCGGACTTTGAATATGTCCTCATTGACAGCACCATATCGAAAGTCCACGCAGATGCGGCGGGCGCAAAAGGGGGGCTGAAGCTGCCTGCATCGGTCGCTCGCGCGGTGGATTGACGACCAAGCTGCATGCTGTTGTCGATGCTATCGGCCTACCGCTGCGAATAAAGCCAACACCCGGCCATTATGGTGACTGTCCGCAAGCTTCAAGCCTTCTATCCGGCTTAGAGGGTGTGGGGCATGTCATTGCTGATGCGGCCTATGATGCCGATCACTTAAGGGCCTTCATTGCCAGCAATCTCAAGGCAACGGCTCAGATCAAGGCCAATCCAACACGTTCCAGTGTCCCAACAATCGACTGGAGGCTGTACAAGGAACGCCATCAGATTGAATGCTTTTTTAACAAGTTGAAACGCTATCGTCGTATTGCGCTGCGATGCGAGAAAACATTGACCGCATTCATGGGCTTCGTCCATCTCGCATGCGCTATGATCTGGTTGCGTTGAATGCAGACACGCCCTAG | IS711 family insertion sequence |
| 155624 | 155624 | -> | 156454 | A |  | Intergenic |
| 156370 | 156375 | -> | 157198 | CTGGTG |  | In frame change in BPI_II171, outer membrane autotransporter (orth: BMI_II170) |
| 157609 | 158400 | -> | 158431 | TGGCGAACGACAACACCGGTGGCGGCACGACGACGGTTGATGCGGGGGCAGGACTTCAGATTGGCACTGGCGGGACGAGCGGCAGCCTTGCGGGCGATATCGCCAACAATGGTGCGCTTGTCGTAAACCGCTCCGACGCACTTAATCTCGCCGGCGCGATCTCCGGCACGGGCAGTCTGACGAAGAACGGTGCTGGCACGCTGACGCTTTCTGGTGCCAACAGCTATACGGGCGCAACGACGGTGTCTGCGGGCACGCTGACGCTAACGGGTGACAATACCGGTGGCGGCACGACGACGGTTGATGTGGGAGCCTTGCTTCAGATTGGCACTGGCGGGACGAGCGGCAGCCTTGCAGGCGATATCGCCAATGATGGTACGCTTGTCGTTGACCGCTCCGACGCGATCGGGCTTAGCGGAGCGATCTCCGGCGCGGGCGGCCTGACGAAGAACGGTGCTGGCACGCTGACGCTTTCTGGTGCCAACAGCTATACGGGCGCAACGACGGTGTCTGCGGGCATACTGACGTTGACGGGTGACAATACCGGTGGCGGCACGACGACGGTTGATGCGAGGGCAGAACTTCATATCGGTACTGGTGGGGCGAGCGGCAGCCTTGCAGGCAATATCGTCAATGATGGTGCGCTTGTCGTTGACCGTAGCGGTGTGATCGGGCTTAGCGGTGTGATCTCCGGCACGGGCGGTCTGATGAAGAGCGGCACAGGCACGGTTACGCTTTCTGGTGTCAACACCTATACGGGTGGCACGACGTTGACGGCGGGCACGCTTGCTG |  | Large in frame change in outer membrane autotransporter BPI_II171 (orth: BM_II170) |
| 160764 | 160764 | -> | 160794 | C |  | Intergenic |
| 165175 | 165175 | -> | 165204 | T |  | Frameshift in BPI_II178, glycolate oxidase, subunit GlcD pseudogene (orth: BMI_II177) |
| 179531 | 179531 | -> | 179559 | A |  | Intergenic |
| 180476 | -> | 180505 | 180652 |  | GTCCATGCCACCCCCGCGCCGCTTTGCGGCGCAGGCATTTTAGTTCATGGGCGCGCTTTATGGACCTCTTAGAAGTCCATGCCGCCCCCGCGCCGCTTTGCGGCGCAGGCATTTTAGTTCATGGGCGCGCTTTATGGACCTCTTAGAA | Intergenic |
| 198831 | 198831 | -> | 199006 | G |  | Intergenic |
| 213745 | 213745 | -> | 213919 | G |  | Frameshift in BPI_II222, CAIB/BAIF family protein pseudogene (orth: BMI_II221) |
| 228604 | 228604 | -> | 228777 | C |  | Frameshift in BPI_II237, malate/L-lactate dehydrogenase family protein pseudogene (orth: BMI_II236) |
| 243750 | 243750 | -> | 243922 | G |  | Intergenic |
| 244826 | -> | 244999 | 245842 |  | GGGCTTGTCTGCATTCAAGGATTCCCTTTTGCATGAAATTCTGATTCAAGGTTGTTGAAGGAGAACAGCCGTGAGCAGACGAAGCCTTACAGATGAGCAATGGAACCGGATCGAAGCATATCTTCCGGGGCGAGTTGGTACGCCCGGCCGCAGTGGCGTTGATAACCGATTATTTGTCGACGCCATCTTGTGGATGGCTGCCAATGCAGCGCACTGGCGCGATCTGCCTGCGACCTTCGGCAAATGGACAGCGGTTCATGCCCGCTTTCGGCGCTGGTCGCACGCCGGTGTATGGGAAAGGCTTTTCCATGCCCTGGCTGATACGCCGGACTTTGAATATGTCCTCATTGATAGCACCATATCGAAAGTCCACGCAGATGCGGCGGGCGCAAAAGGGGGGCTGAAGCTGCCTGCATCGGTCGCTCGCGCGGTGGATTGACGACCAAGCTGCATGCTGTTGTCGATGCTATCGGCCTACCGCTGCGAATAAAGCCAACACCCGGCCATTATGGTGACTGTCCGCAAGCTTCAAGCCTTCTATCCGGCTTAGAGGGTGTGGGGCATGTCATTGCTGATGCGGCCTATGATGCCGATCACTTAAGGGCCTTCATTGCCAGCAATCTCAAGGCAACGGCTCAGATCAAGGCCAATCCAACACGTTCCAGTGTCCCAACAATCGACTGGAGGCTGTACAAGGAACGCCATCAGATTGAATGCTTTTTTAACAAGTTGAAACGCTATCGTCGTATTGCGCTGCGATGCGAGAAAACATTGACCGCATTCATGGGCTTCGTCCATCTCGCATGCGCTATGATCTGGTTGCGTTGAATGCAGACACGCCCTA | IS711 family insertion sequence |
| 258986 | -> | 260003 | 260003 |  | A | Frameshift in BPI_II272, nitrous oxide reductase regulatory protein NosR pseudogene (orth: BMI_II269). |
| 261906 | 261909 | -> | 262922 | ATTT |  | Frameshift in BPI_II274, copper ABC transporter, periplasmic copper-binding protein NosD pseudogene (orth: BMI_II271). |
| 267708 | 267708 | -> | 268720 | G |  | Frameshift in BPI_II281, NnrS family protein, uncharacterized protein involved in response to NO, pseudogene (orth: BMI_II278). |
| 274520 | 274530 | -> | 275531 | TGATTGCCTGC |  | Change beginning of BPI_II290, 3-octaprenyl-4-hydroxybenzoate carboxy-lyase, maybe a pseudogene (orth: BMI_II286). |
| 287805 | -> | 288807 | 288807 |  | A | Intergenic |
| 296020 | 296020 | -> | 297021 | T |  | Intergenic |
| 309794 | -> | 310796 | 310806 |  | CAAGCGGTGGA | Frameshift in BPI_II326, spermidine/putrescine ABC transporter membrane protein, putative, which is pseudogene BMI_II323 in B. microti. |
| 323995 | 323995 | -> | 325007 | G |  | Intergenic |
| 323766 | -> | 324779 | 324779 |  | C | Frameshift in BPI_II340, acid-resistance protein, different from orthologs in other Brucella . |
| 331616 | -> | 332628 | 332633 |  | CGCGCC | In frame change in BPI_II348, hypothetical protein (orth: BMI_II345). |
| 336328 | 336336 | -> | 337344 | CGCTGCCGC |  | In frame change in BPI_II351, cytochrome c heme-binding site (orth: BMI_II348). |
| 336537 | -> | 337546 | 337546 |  | G | Intergenic |
| 339917 | 339931 | -> | 340925 | CTCGCGCTGATTGCG |  | In frame change in BPI_II355, disulfide bond formation protein DsbB (orth: BMI_II352). |
| 344671 | 363011 | -> | 345664 | CAGACTGGCACCAAAGAGCATTAAAACACCCATAAGCCCGCGT up to AGAGTGGGAAATCCCGCGTCCGGCA |  | 18 kbp fragment not present in B. pinnipedialis, see discussion in the text. Only part of the insert is shown here. |
| 372907 | -> | 355561 | 356404 |  | GGCTTGTCTGCATTCAAGGATTCCCTTTTGCATGAAATTCTGATTCAAGGTTGTTAAAGGAGAACAGCCGTGAGCAGACGAAGCCTTACAGATGAGCAATGGAACCGGATCGAAGCATATCTTCCGGGGCGAGTTGGTACGCCCGGCCGCAGTGGCGTCGATAACCGATTATTTGTCGACGCCATCTTGTGGATGGCTGCCAATGCAGCGCACTGGCGCGATCTGCCTGCGACCTTCGGCAAATGGACAGCGGTTCATGCCCGCTTTCGGCGCTGGTCGCACGCCGGTGTATGGGAAAGGCTTTTCCATGCCCTGGCTGATACGCCGGACTTTGAATATGTCCTCATTGATAGCACCATATCGAAAGTCCACGCAGATGCGGCGGGCGCAAAAGGGGGGCTGAAGCTGCCTGCATCGGTCGCTCGCGCGGTGGATTGACGACCAAGCTGCATGCTGTTGTCGATGCTATCGGCCTACCGCTGCGAATAAAGCCAACACCCGGCCATTATGGTGACTGTCCGCAAGCTTCAAGCCTTCTATCCGGCTTAGAGGGTGTGGGGCATGTCATTGCTGATGCGGCCTATGATGCCGATCACTTAAGGGCCTTCATTGCCAGCAATCTCAAGGCAACGGCTCAGATCAAGGCCAATCCAACACGTTCCAGTGTCCCAACAATCGACTGGAGGCTGTACAAGGAACGCCATCAGATTGAATGCTTTTTTAACAAGTTGAAACGCTATCGTCGTATTGCGCTGCGATGCGAGAAAACATTGACCGCATTCATGGGCTTCGTCCATCTCGCATGCGCTATGATCTGGTTGCGTTGAATGCAGACACGCCCTAG | IS711 family insertion sequence. At this location, orthologs of genes BPI_II373-4 are deleted. |
| 384280 | 384280 | -> | 367776 | A |  | Frameshift in BPI_II382, branched-chain amino acid ABC transporter, periplasmic amino acid-binding protein, pseudogene (orth: BMI_II397). |
| 396852 | 396852 | -> | 380347 | T |  | Frameshift in BPI_II393, ROK family protein pseudogene (orth: BMI_II408). |
| 431530 | -> | 415026 | 415026 |  | G | Frameshift in BPI_II424, uracil-xanthine permease pseudogene (orth: BMI_II439). |
| 449459 | -> | 432956 | 432997 |  | TGCAGCGGGCTGATCTGATAAGCGCGACTGGGAGGAGGAGTG | Intergenic |
| 457855 | -> | 441394 | 441394 |  | T | Frameshift in BPI_II453, transporter pseudogene (orth: BMI_II468). |
| 463456 | -> | 446996 | 447033 |  | CACTAGGGCGTGTCTGCATTCAACGCAACCAGATCATA | Changes the end of BPI_II459, hypothetical protein pseudogene, shorter than B. microti counterpart (BMI_II474) |
| 463459 | -> | 447038 | 447041 |  | ATGC | IS711 family insertion sequence |
| 463467 | -> | 447050 | 447218 |  | CGAAGCCCATGAATGCGGTCAATGTTTTCTCGCATCGCAGCGCAATACGACGATAGCGTTTCAACTTGTTAAAAAAGCATTCAATCTGATGGCGTTCCTTGTACAGCCTCCAGTCGATTGTTGGGACACTGGAACGTGTTGGATTGGCCTTGATCTGAGCCGTTGCCTT |  |
| 463473 | -> | 447225 | 447301 |  | GCTGGCAATGAAGGCCCTTAAGTGATCGGCATCATAGGCCGCATCAGCAATGACATGCCCCACACCCTCTAAGCCGG |  |
| 463483 | -> | 447312 | 447650 |  | TGAAGCTTGCGGACAGTCACCATAATGGCCGGGTGTTGGCTTTATTCGCAGCGGTAGGCCGATAGCATCGACAACAGCATGCAGCTTGGTCGTCAATCCACCGCGCGAGCGACCGATGCAGGCAGCTTCAGCCCCCCTTTTGCGCCCGCCGCATCTGCGTGGACTTTCGATATGGTGCTATCAATGAGGACATATTCAAAGTCCGGCGTATCAGCCAGGGCATGGAAAAGCCTTTCCCATACACCGGCGTGCGACCAGCGCCGAAAGCGGGCATGAACCGCTGTCCATTTGCCGAAGGTCGCAGGCAGATCGCGCCAGTGCGCTGCATTGGCAGCCATC |  |
| 463490 | -> | 447659 | 447837 |  | GGCGTCGACAAATAATCGGTTATCGACGCCACTGCGGCCGGGCGTACCAACTCGCCCCGGAAGATATGCTTCGATCCGGTTCCATTGCTCATCTGTAAGGCTTCGTCTGCTCAAGGCTGTTCTCCTTCAACAACCTTGAATCAGAATTTCATGCAAAAGGGAATCCTTGAATGCAGACA |  |
| 470409 | 470409 | -> | 454755 | C |  | Frameshift in BPI_II470, glycosyl hydrolase, family 25 pseudogene (orth: BMI_II483). |
| 508087 | 508087 | -> | 492432 | C |  | Difference in the beginning of BPI_II509, SURF1 protein (orth: BMI_II522). |
| 514197 | -> | 498543 | 499386 |  | GGCGTGTCTGCATTTAACGTAACCAGATCATAGCGCATGCGAGATGGACGAAACCCATGAATGCGGTCAATGTTTTCTCGCATCGCAGCGCAATACGACGATAGCGTTTCAACTTGTTAAAAAAGCATTCAATCTGATGGCGTTCCTTGTACAGCCTCCAGTCGATTGTTGGGACACTGGAACGTGTTGGATTGGCCTTGATCTGAGCCGTTGCCTTGAGATTGCTGGCAATGAAGGCCCTTAAGTGATCGGCATCATAGGCCGCATCAGCAATGACATGCCCCACACCCTCTAAGCCGGATAGAAGGCTTGAAGCTTGCGGACAGTCACCATAATGGCCGGGTGTTGGCTTTATTCGCAGCGGTAGGCCGATAGCATCGACAACAGCATGCAGCTTGGTCGTCAATCCACCGCGCGAGCGACCGATGCAGGCAGCTTCAGCCCCCCTTTTGCGCCCGCCGCATCTGCGTGGACTTTCGATATGGTGCTATCAATGAGGACATATTCAAAGTCCGGCGTATCAGCCAGGGCATGGAAAAGCCTTTCCCATACACCGGCGTGCGACCAGCGCCGAAAGCGGGCATGAACCGCTGTCCATTTGCCGAAGGTCGCAGGCAGATCGCGCCAGTGCGCTGCATTGGCAGCCATCCACAAGATGGCGTCGACAAATAATCGGTTATCGACGCCACTGCGGCCGGGCGTACCAACTCGCCCCGGAAGATATGCTTCGATCCGGTTCCATTGCTCATCTGTAAGGCTTCGTCTGCTCACGGCTGTTCTCCTTTAACAACCTTGAATCAGAATTTCATGCAAAAGGGAATCCTTGAATGCAGACAAGCCCTAG | IS711 family insertion sequence |
| 522546 | -> | 507736 | 508579 |  | GGGCTTGTCTGCATTCAAGGATTCCCTTTTGTACGAAATTCTGATTCAAGGTTGTTAAAGGAGAACAGCCGTGAGCAGACGAAGCCTTACAGATGAGCAATGGAACCGGATCGAAGCATATCTTCCGGGGCGAGTTGGTACGCCCGGCCGCAGTGGCGTCGATAACCGATTATTTGTCGACGCCATCTTGTGGATGGCTGCCAATGCAGCGCACTGGCGCGATCTGCCTGCGACCTTCGGCAAATGGACAGCGGTTCATGCCCGCTTTCGGCGCTGGTCGCACGCCGGTGTATGGGAAAGGCTTTTCCATGCCCTGGCTGATACGCCGGACTTTGAATATGTCCTCATTGACAGCACCATATCGAAAGTCCACGCAGATGCGGCGGGCGCAAAAGGGGGGCTGAAGCTGCCTGCATCGGTCGCTCGCGCGGTGGATTGACGACCAAGCTGCATGCTGTTGTCGATGCTATCGGCCTACCGCTGCGAATAAAGCCAACACCCGGCCATTATGGTGACTGTCCGCAAGCTTCAAGCCTTCTATCCGGCTTAGAGGGTGTGGGGCATGTCATTGCTGATGCGGCCTATGATGCCGATCACTTAAGGGCCTTCATTGCCAGCAATCTCAAGGCAACGGCTCAGATCAAGGCCAATCCAACACGTTCCAGTGTCCCAACAATCGACTGGAGGCTGTACAAGGAACGCCATCAGATTGAATGCTTTTTTAACAAGTTGAAACGCTATCGTCGTATTGCGCTGCGATGCGAGAAAACATTGACCGCATTCATGGGCTTCGTCCATCTCGCATGCGCTATGATCTGGTTGCGTTGAATGCAGACACGCCCTA | IS711 family insertion sequence |
| 531773 | 531778 | -> | 517805 | AGAAGG |  | Intergenic |
| 531810 | 531810 | -> | 517836 | T |  | Frameshift shorten BPI_II535, helix-turn-helix, AraC type (orth: bMI_II544). |
| 531875 | -> | 517902 | 517902 |  | A | Frameshift shorten BPI_II535, helix-turn-helix, AraC type (orth: bMI_II544). |
| 531910 | -> | 517938 | 517943 |  | CTTCTT | In frame change in BPI_II535. |
| 533467 | 533467 | -> | 519499 | G |  | Frameshift in BPI_II536, IS711 family insertion sequence transposase OrfB, pseudogene in B. microti (BMI_II545). |
| 534103 | -> | 520136 | 587524 |  | AGCCCTAGCTTGAAGTGCGACTTGCAAGCGATA up to CGCTCGGAACGGCGGGAATTGAATCAGCTGTC | 67kb region not present in B. microti. Only a fragment of the insert is shown here. |
| 537481 | -> | 590903 | 590912 |  | GGAATCGACT | Frameshift in BPI_II608, transposase IS66, orf3, B. microti ortholog (BMI_II549) is shorter than in most other Brucella. |
| 539760 | 539760 | -> | 593190 | C |  | Frameshift in BPI_II611, partial Isbm1 transposase orfA, which is pseudogene BMI_II552 in B. microti. |
| 540537 | 540537 | -> | 593966 | T |  | Intergenic |
| 543488 | -> | 596918 | 596918 |  | T | Intergenic |
| 599436 | -> | 652867 | 652869 |  | CGC | In frame change in BPI_II665, response regulator PleD (orth: BMI_II606). |
| 607521 | -> | 660955 | 660955 |  | T | Intergenic |
| 626790 | 626790 | -> | 680223 | T |  | Frameshift in BPI_II699, alpha/beta hydrolase PcaL, pseudogene BMI_II640 in B. microti. |
| 627864 | -> | 681298 | 681298 |  | C | Frameshift in BPI_II701, intradiol ring-cleavage dioxygenase PcaH, pseudogene BMI_II642 in B. microti. |
| 630851 | -> | 684286 | 684286 |  | A | Frameshift in BPI_II704, amino acid ABC transporter, periplasmic amino-acid binding protein, pseudogene BMI_II645 in B. microti. |
| 633069 | 633070 | -> | 686503 | GG |  | Frameshift in BPI_II706, branched-chain amino acid ABC transporter, permease protein pseudogene, ortholog BMI_II647 intact in B. microti. |
| 637898 | 637898 | -> | 691330 | A |  | Intergenic |
| 637964 | -> | 691397 | 692240 |  | GGGCGTGTCTGCATTCAACGCAACCAGATCATAGCGCATGCGAGATGGACGAAGCCCATGAATGCGGTCAATGTTTTCTCGCATCGCAGCGCAATACGACGATAGCGTTTCAACTTGTTAAAAAAGCATTCAATCTGATGGCGTTCCTTGTACAGCCTCCAGTCGATTGTTGGGACACTGGAACGTGTTGGATTGGCCTTGATCTGAGCCGTTGCCTTGAGATTGCTGGCAATGAAGGCCCTTAAGTGATCGGCATCATAGGCCGCATCAGCAATGACATGCCCCACACCCTCTAAGCCGGATAGAAGGCTTGAAGCTTGCGGACAGTCACCATAATGGCCGGGTGTTGGCTTTATTCGCAGCGGTAGGCCGATAGCATCGACAACAGCATGCAGCTTGGTCGTCAATCCACCGCGCGAGCGACCGATGCAGGCAGCTTCAGCCCCCCTTTTGCGCCCGCCGCATCTGCGTGGACTTTCGATATGGTGCTATCAATGAGGACATATTCAAAGTCCGGCGTATCAGCCAGGGCATGGAAAAGCCTTTCCCATACACCGGCGTGCGACCAGCGCCGAAAGCGGGCATGAACCGCTGTCCATTTGCCGAAGGTCGCAGGCAGATCGCGCCAGTGCGCTGCATTGGCAGCCATCCACAAGATGGCGTCGACAAATAATCGGTTATCGACGCCACTGCGGCCGGGCGTACCAACTCGCCCCGGAAGATATGCTTCGATCCGGTTCCATTGCTCATCTGTAAGGCTTCGTCTGCTCACGGCTGTTCTCCTTTAACAACCTTGAATCAGAATTTCATGCAAAAGGGAATCCTTGAATGCAGACAGGCCCTA | IS711 family insertion sequence |
| 643640 | -> | 697917 | 698426 |  | CATCGCCGCGGTCGCGGCAACCGCTTTCAGCATCATGCCGGTGGCTGTCGCCTTTTTCGCAGCCGCAGTCGCCATGCTGGTTTTCAAGGTGATCCCGCTCAATGAGGTCTATGACGAGATCGACGGCCCGATTCTGGTCATGCTGGCCGCGCTCATTCCCGTTTCGGATGCGCTGAGAACCACAGGCGGCACGGAGATTATCGCGCAAGGTCTCGCCTCGCTCGGGCAGCAATTGCCGCCCGCAGGCGCGCTGGCGCTGATATTGGTTGCGGCCATGATGGTGACGCCATTTCTCAACAATGCCGCGACCGTGCTGGTTATGGCCCCCATCGCCACCAGTTTTGCGGCCGGGCTCGGCTTCAGGCCGGAAGCCTTTCTCATGGCCGTCGCCATCGGGGCGGGCTGCGATTTTCTCACCCCCATCGGCCATCAATGCAACACGCTGGTCATGGGGCCGGGTGGCTACAAATTCAGCGATTATCCCCGGCTTGGCCTGCCGCTTTCCATCAT | In BPI_II717, TrkA family protein. The gene is apparently intact in B. pinnipedialis and shows a deletion in B. microti. |
| 650187 | 650187 | -> | 704973 | C |  | Frameshift in BPI_II727, protein of unknown function DUF81, quite different from the ortholog BMI_II666. |
| 649848 | -> | 704635 | 704635 |  | A | Frameshift in BPI_II727, protein of unknown function DUF81, quite different from the ortholog BMI_II666. |
| 654634 | 654634 | -> | 709419 | C |  | Frameshift in BPI_II732, iron compound ABC transporter, periplasmic iron compound-binding protein, changing the beginning of gene (orth: BMI_II671). |
| 663804 | -> | 718590 | 718601 |  | CTTTGCATGTTC | Intergenic |
| 669484 | -> | 724282 | 724282 |  | T | Frameshift in BPI_II743, hypothetical protein, pseudogene BMI_II683 in B. microti. |
| 673339 | 673339 | -> | 728136 | T |  | Frameshift in BPI_II748, sugar ABC transporter, periplasmic sugar-binding protein pseudogene (orth: BMI_II687). |
| 674120 | 674120 | -> | 728915 | C |  | Frameshift in BPI_II748, sugar ABC transporter, periplasmic sugar-binding protein pseudogene (orth: BMI_II687). |
| 681096 | -> | 735892 | 735893 |  | GC | Frameshift in BPI_II756, ABC transporter related protein, pseudogene BMI_II695 in B. microti. |
| 689236 | 689266 | -> | 744032 | AATATCGATTTTAACGATTAAAGCGATTATT |  | Intergenic |
| 692492 | 692495 | -> | 747257 | TATT |  | Intergenic |
| 693911 | -> | 748674 | 748716 |  | GGAAGCCGCCGTTTTGAAGGCGCTGCACCTTGCGCGCGAAAAC | Change in BPI_II768, myo-inositol catabolism IolC protein pseudogene, also pseudogene (BMI_II707) in B. microti. |
| 704224 | 704224 | -> | 759028 | T |  | Intergenic |
| 709421 | 709444 | -> | 764224 | GCTATGATGACCGCCGTCGACGCC |  | Intergenic |
| 715059 | -> | 769840 | 769845 |  | CCCCCC | Intergenic |
| 730294 | -> | 785081 | 785082 |  | AA | Frameshift in BPI_II800, oxidoreductase, short chain dehydrogenase/reductase family pseudogene (orth: BMI_II739). |
| 736271 | -> | 791060 | 791060 |  | A | Intergenic |
| 750655 | 750734 | -> | 805443 | GGGAGTAAGGGAGTAAGGGAGTAAGGGAGTAAGGGAGTAAGGGAGTAAGGGAGTAAGGGAGTAAGGGAGTAAGGGAGTAA |  | Intergenic |
| 754055 | -> | 808765 | 809594 |  | CTCTGATCCTGAAGCCATCCTAGGGCTTGTCTGCATTCAAGGATTCCCTTTTGTACGAAATTCTGATTCAAGGTTGTTAAAGGAGAACAGCCTTGAGCAGACGAAGCCTTACAGATGAGCAATGGAACCGGATCGAAGCATATCTTCCGGGGCGAGTTGGTACGCCCGGCCGCAGTGGCGTCGATAACCGATTATTTGTCGACGCCATCTTGTGGATGGCTGCCAATGCAGCGCACTGGCGCGATCTGCCTGCGACCTTCGGCAAATGGACAGCGGTTCATGCCCGCTTTCGGCGCTGGTCGCACGCCGGTGTATGGGAAAGGCTTTTCCATGCCCTGGCTGATACGCCGGACTTTGAATATGTCCTCATTGATAGCACCATATCGAAAGTCCACGCAGATGCGGCGGGCGCAAAAGGGGGGCTGAAGCTGCCTGCATCGGTCGCTCGCGCGGTGGATTGACGACCAAGCTGCATGCTGTTGTCGATGCTATCGGCCTACCGCTGCGAATAAAGCCAACACCCGGCCATTATGGTGACTGTCCGCAAGCTTCAAGCCTTCTATCCGGCTTAGAGGGTGTGGGGCATGTCATTGCTGATGCGGCCTATGATGCCGATCACTTAAGGGCCTTCATTGCCAGCAATCTCAAGGCAACGGCTCAGATCAAGGCCAATCCAACACGTTCCAGTGTCCCAACAATCGACTGGAGGCTGTACAAGGAACGCCATCAGATTGAATGCTTTTTTAACAAGTTGAAACGCTATCGTCGTATTGCGCTGCGATGCGAGAAAACATTGACCGCATTCATGGGCTTCGTCCATCTCGCATGCG | IS711 family insertion sequence |
| 754070 | -> | 809610 | 809623 |  | GTTGAATGCAGACA | Intergenic |
| 775406 | -> | 830960 | 830961 |  | CT | Frameshift in BPI_II849, peptidase, S24 family pseudogene, (orth: BMI_II786). |
| 778598 | 778598 | -> | 834152 | A |  | Frameshift in BPI_II851, acetyl-CoA acetyltransferase pseudogene (orth: BMI_II788). |
| 834260 | -> | 889815 | 889815 |  | C | Frameshift in BPI_II904, IS711 transposase OrfB pseudogene (orth: BMI_II841). |
| 846292 | 846292 | -> | 901846 | G |  | Frameshift in BPI_II916, ribose ABC transporter, permease protein pseudogene (orth: BMI_II853). |
| 874655 | -> | 930210 | 930210 |  | G | Frameshift in BPI_II946, methylated-DNA-[protein]-cysteine S-methyltransferase, which is pseudogene BMI_II883 in B. microti. |
| 880347 | 880361 | -> | 935901 | GACTCCATCGACAGG |  | In frame change in BPI_II955, sec-independent protein translocase TatC (orth: BMI_II892). |
| 883669 | 883669 | -> | 939208 | A |  | Frameshift in BPI_II959, oxidoreductase, FMN-binding pseudogene (orth: BMI_II896). |
| 909637 | 909637 | -> | 965175 | C |  | Frameshift in BPI_II980, alanine racemase pseudogene (orth: BMI_II917). |
| 914103 | -> | 969642 | 969642 |  | T | Intergenic |
| 933827 | 933827 | -> | 989365 | C |  | Frameshift in BPI_II1001, transcriptional regulator, GntR family pseudogene (orth: BMI_II938). |
| 942239 | 942259 | -> | 997776 | TTTGCCGGAATGGACCGGCAC |  | In frame change in BPI_II1007, hypothetical protein (orth: BMI_II944) |
| 944983 | 945053 | -> | 1000499 | CACGCTCGCTGAAGTGCACGGGCTTGGACTTGAAGCCGCTCAAGGATTGACACTGACGGAAGGCTTCTACT |  | Frameshift in BPI_II1010, branched-chain amino acid ABC transporter, periplasmic amino acid-binding protein pseudogene (orth: BMI_II947). |
| 948110 | -> | 1003557 | 1003557 |  | G | Frameshift in BPI_II1014, branched-chain amino acid ABC transporter, permease protein (orth: BMI_II951). |
| 952384 | 952384 | -> | 1007830 | A |  | Frameshift in BPI_II1018, ABC transporter, ATP-binding protein pseudogene (orth: BMI_II955). |
| 960325 | 960340 | -> | 1015770 | GAGTAAGGGAGTAAGG |  | Intergenic |
| 975840 | -> | 1031271 | 1031378 |  | CATCACCATGGGCATCATCACCATGGGCATGACCATCACCATCATGATCATCACGATCACGACCATGTTTGCGGGCCGGACTGCGACCACGATCACGATCACGATCAT | In frame change in BPI_II1043, cobalamin synthesis protein/P47K family protein (orth: BMI_II980). |
| 977366 | 977381 | -> | 1032919 | TTACTGCCCTACTGCC |  | Intergenic |
| 977267 | -> | 1032806 | 1032821 |  | AGGGCAGTAGGGCAGT | Intergenic |
| 983425 | 983425 | -> | 1038962 | G |  | Frameshift in BPI_II1051, ribose ABC transporter, ATP-binding protein pseudogene (orth: BMI_II988). |
| 997813 | -> | 1053350 | 1053352 |  | CCC | In frame change in BPI_II1063, glutamyl-tRNA(gln) amidotransferase subunit A pseudogene, also pseudogene BMI_II1000 in B. microti. |
| 998275 | -> | 1053815 | 1053815 |  | C | Frameshift in BPI_II1063, glutamyl-tRNA(gln) amidotransferase subunit A pseudogene, also pseudogene BMI_II1000 in B. microti. |
| 1009287 | 1009323 | -> | 1064826 | AAGCGGTTATTCTTCTGCATCCTCGCTTTGCTGGCGC |  | Frameshift in BPI_II1075, hypothetical protein pseudogene (orth: BMI_II1012). |
| 1015115 | 1015142 | -> | 1070617 | GTAATAAAAGAAGCACGCTTATCGTGCC |  | End of BPI_II1082, acetylglutamate kinase (orth: BMI_II1019), don't change the reading frame. |
| 1025356 | -> | 1080832 | 1080838 |  | TCCCCCC | Intergenic |
| 1038883 | 1050624 | -> | 1094364 | AAATTCCGTTTTTCTACGTATCTACCAATA up to GCATTTTGGAAGCCTCTTCTGGCACCA |  | 11kbp fragment specific of B. microti. |
| 1052790 | -> | 1096531 | 1096531 |  | T | Intergenic |
| 1061628 | 1061646 | -> | 1105368 | GTTTAACCAAGAAGGCTGG |  | Between BPI_II1120 (orth: BMI_II1063), peptide chain release factor 3, and BPI_II1121 (orth: BMI_II1064), phosphopantothenoylcysteine synthase/decarboxylase. This fragment do not modify the reading frames. |
| 1068199 | 1068200 | -> | 1111920 | GA |  | Intergenic |
| 1077296 | 1077296 | -> | 1121015 | A |  | Intergenic |
| 1079329 | -> | 1123049 | 1123049 |  | A | Frameshift in BPI_II1141, branched-chain alpha-keto acid dehydrogenase subunit E2, which is pseudogene BMI_II1084 in B. microti. |
| 1082391 | 1085271 | -> | 1126110 | CCGGAAAATTCGCTGCCATCTCCCAAAATCC up to CAGCAGCCAACGCGGGCATAAGCGCGATTT |  | 2.8 kbp fragment containing BMI_II1086, 2-oxoisovalerate dehydrogenase alpha and beta subunit and BMI_II1087, 3-hydroxybutyryl-CoA dehydrogenase, deleted from B. pinnipedialis, where we have annotated pseudogenes BPI_II1143 and BPI_II1144. |
| 1096353 | 1096355 | -> | 1137191 | CAA |  | Intergenic |
| 1115255 | 1115497 | -> | 1156090 | AAAGCAGATGATGTTCGTAACCATCTCGGAAACTGTTGAGCCTGGTTGGCAGATGATCCGGCAACAGACCGGTTACGAATTGCAAAAGGCGATCGCTGATTGCGCTGCCGAGACGAAGCTTTTCGGTTATGCCGTCAAAACGGCTTTTGATGCTGAAAGCCAAGGGGATCTTGTCTGTGCCAAAAGCTTGAAGGAATAGGAAGAGGTCCTTGCCGTAGCGTCGTGACAAATCATAAGCTGAGC |  | Intergenic |
| 1120053 | -> | 1160646 | 1160659 |  | GTGCTGTTCTCGCT | 23S ribosomal rna BPI_II1176 |
| 1120063 | -> | 1160670 | 1160677 |  | ACGCATTC |  |
| 1120090 | 1120103 | -> | 1160703 | CAATAAGGCAATAT |  |  |
| 1120116 | 1120117 | -> | 1160715 | CT |  |  |
| 1120137 | -> | 1160736 | 1160736 |  | G |  |
| 1120150 | -> | 1160750 | 1160766 |  | CCATCCAGCGTTGCTCC |  |
| 1123355 | 1123356 | -> | 1163970 | TA |  | Frameshift in BPI_II1180, N-acetylglucosamine kinase, pseudogene BMI_II1124 in B. microti. |
| 1129995 | -> | 1170610 | 1170611 |  | GC | Frameshift in BPI_II1188, hypothetical protein, pseudogene BMI_II1132 in B. microti. |
| 1138364 | 1138364 | -> | 1178979 | C |  | Frameshift in BPI_II1200, flagellar hook-associated protein FlgK pseudogene (orth: BMI_II1144). |
| 1171131 | 1171131 | -> | 1211745 | T |  | Frameshift in BPI_II1220, 2-keto-4-pentenoate hydratase (hpa operon) pseudogene (orth: BMI_II1164). |
| 1180872 | 1180880 | -> | 1221485 | ATATCCACC |  | In frame change in BPI_II1230, hypothetical protein (orth: BMI_II1174). |
| 1207416 | -> | 1248022 | 1248022 |  | T | Intergenic |
| 1210668 | 1210668 | -> | 1251273 | A |  | Frameshift in BPI_II1256, branched-chain amino acid ABC transporter, permease protein pseudogene (orth: BMI_II1200). |
| 1211856 | -> | 1252462 | 1252463 |  | CA | Frameshift in BPI_II1257, high-affinity branched-chain amino acid transport system permease protein pseudogene (orth: BMI_II1201). |
| 1213752 | -> | 1254360 | 1254360 |  | C | Frameshift in BPI_II1260, cadmium-translocating P-type ATPase pseudogene (orth: BMI_II1204). |
| 1217670 | 1217670 | -> | 1258277 | A |  | Intergenic |
