# Supplementary material for: The genome sequence of Brucella pinnipedialis B2/94 sheds light on the evolutionary history of the genus Brucella
Source: BMC Evol Biol. 2011 Jul 11;11:200. doi: 10.1186/1471-2148-11-200 (PMC3146883; doi:10.1186/1471-2148-11-200)
Supplement: Additional file 3 — Occurrences of the largest genome fragments unshared between B. pinnipedialis B2/94 and B. microti CCM 4915 in selected Brucella genomes. Occurrences of the largest genome fragments unshared between B. pinnipedialis B2/94 and B. microti CCM 4915 in selected Brucella genomes. For each organism, the number of positions in its genome that are found similar to the original fragment is reported. The column "Origin of sequence data" indicates "GenBank" for sequences deposited in the complete genome division of GenBank, "Patric" if the sequence originates from the Pathosystems Resource Integration Center of the Virginia Bioinformatics Institute, and "Broad" if he sequence originates from the Brucella genome sequencing project at the Broad Institute. [file 1471-2148-11-200-S3.DOC]

|  |  | **# of positions of the insert matched at e=1.e-100** | | | | | |
| --- | --- | --- | --- | --- | --- | --- | --- |
| **Species name** | **Origin of sequence data** | **67kbp** | **18kbp** | **21kbp** | **11kbp** | **2.8kbp** | **2.6kbp** |
| *Brucella* sp. NF2653 | Patric | 4566 | 0 | 942 | 0 | 0 | 2653 |
| *Brucella* sp. 83/13 | Broad | 4924 | 0 | 1203 | 0 | 0 | 2653 |
| *B. inopinata* B01 | Patric | 4984 | 0 | 847 | 0 | 0 | 2653 |
| *Brucella* sp. B02 | Patric | 4455 | 1191 | 840 | 0 | 0 | 2653 |
| *B. microti* CCM 4915 | GenBank | 5358 | 18341 | 849 | 11742 | 2881 | 2653 |
| *B. neotomae* 5K33 | Broad | 5715 | 18195 | 21117 | 0 | 2881 | 2653 |
| *B. ceti* M13/05/1 | Broad | 4785 | 18291 | 21434 | 0 | 2881 | 0 |
| *B. ceti* M644/93/1 | Broad | 4122 | 18291 | 21406 | 0 | 2881 | 0 |
| *B. pinnipedialis* M163/99/10 | Broad | 2950 | 0 | 20851 | 0 | 2881 | 0 |
| *B. pinnipedialis* M292/94/1 | Broad | 66385 | 0 | 664 | 0 | 0 | 0 |
| *B. pinnipedialis* B2/94 | GenBank | 65371 | 0 | 20864 | 0 | 0 | 0 |
| *Brucella* sp. F5/99 | Broad | 64478 | 18341 | 0 | 0 | 2881 | 0 |
| *B. ceti* cudo | Patric | 67122 | 18341 | 852 | 0 | 2881 | 0 |
| *B. ceti* M490/95/1 | Broad | 3466 | 18202 | 0 | 0 | 2881 | 0 |
| *B. ceti* B1/94 | Broad | 3549 | 18202 | 198 | 0 | 2881 | 0 |
| *B. ovis* ATCC 25840 | GenBank | 29997 | 0 | 21617 | 0 | 0 | 0 |
| *Brucella* sp. NVSL 07-0026 | Broad | 5359 | 18341 | 853 | 0 | 0 | 0 |
| *B. abortus* Tulya (biovar 3) | Broad | 4804 | 0 | 21659 | 0 | 2881 | 0 |
| *B. abortus* C68 (biovar 9) | Broad | 3371 | 0 | 20872 | 0 | 2881 | 0 |
| *B. abortus* B3196 (biovar 5) | Broad | 5345 | 10862 | 21713 | 0 | 2881 | 2652 |
| *B. abortus* 870 (biovar 6) | Broad | 4595 | 0 | 21484 | 0 | 2881 | 0 |
| *B. abortus* 292 (biovar 4) | Broad | 4646 | 0 | 21675 | 0 | 2881 | 0 |
| *B. abortus* 86/8/59 (biovar 2) | Broad | 4993 | 0 | 21680 | 0 | 2881 | 0 |
| *B. abortus* 9-941 (biovar 1) | GenBank | 5356 | 0 | 21713 | 0 | 2881 | 0 |
| *B. abortus* NCTC 8038 | Broad | 4783 | 0 | 21579 | 0 | 2881 | 0 |
| *B. abortus* S19 (biovar 1) | GenBank | 5356 | 0 | 21713 | 0 | 2881 | 0 |
| *B. abortus* 2308 | GenBank | 5363 | 0 | 21713 | 0 | 2881 | 0 |
| *B. melitensis* Ether (biovar 3) | Broad | 3040 | 0 | 20872 | 0 | 2881 | 0 |
| *B. melitensis* ATCC 23457 | GenBank | 5360 | 0 | 21713 | 0 | 2881 | 0 |
| *B. melitensis* 63/9 (biovar 2) | Broad | 3550 | 0 | 20872 | 0 | 2881 | 0 |
| *B. melitensis* Rev.1 (biovar 1) | Broad | 4819 | 0 | 21542 | 0 | 2881 | 0 |
| *B. melitensis* 16M (biovar 1) | GenBank | 5357 | 0 | 21713 | 0 | 2881 | 0 |
| *B. suis* 513 (biovar 5) | Broad | 4166 | 0 | 21434 | 0 | 2881 | 2653 |
| *B. suis* 1330 (biovar 1) | GenBank | 5356 | 18291 | 847 | 0 | 2881 | 2653 |
| *B. suis* 686 (biovar 3) | Broad | 4696 | 18291 | 826 | 0 | 2881 | 2653 |
| *B. canis* ATCC 23365 | GenBank | 5356 | 18152 | 847 | 0 | 2881 | 2653 |
| *B. suis* 40 (biovar 4) | Broad | 4212 | 18152 | 585 | 0 | 2881 | 2653 |
| *B. suis* ATCC 23445 (biovar 2) | GenBank | 5413 | 18193 | 21713 | 0 | 2881 | 2653 |
