# Supplementary material for: The genome sequence of Brucella pinnipedialis B2/94 sheds light on the evolutionary history of the genus Brucella
Source: BMC Evol Biol. 2011 Jul 11;11:200. doi: 10.1186/1471-2148-11-200 (PMC3146883; doi:10.1186/1471-2148-11-200)
Supplement: Additional file 4 — Evolutionary history of the unshared sequence fragments along the Brucella evolutionary tree. Evolutionary history of the unshared sequence fragments along the Brucella evolutionary tree. The tree is represented as a cladogram with the same topology as that of the whole gene tree (Figure 1). Plots are generated using Mesquite. [file 1471-2148-11-200-S4.PDF]

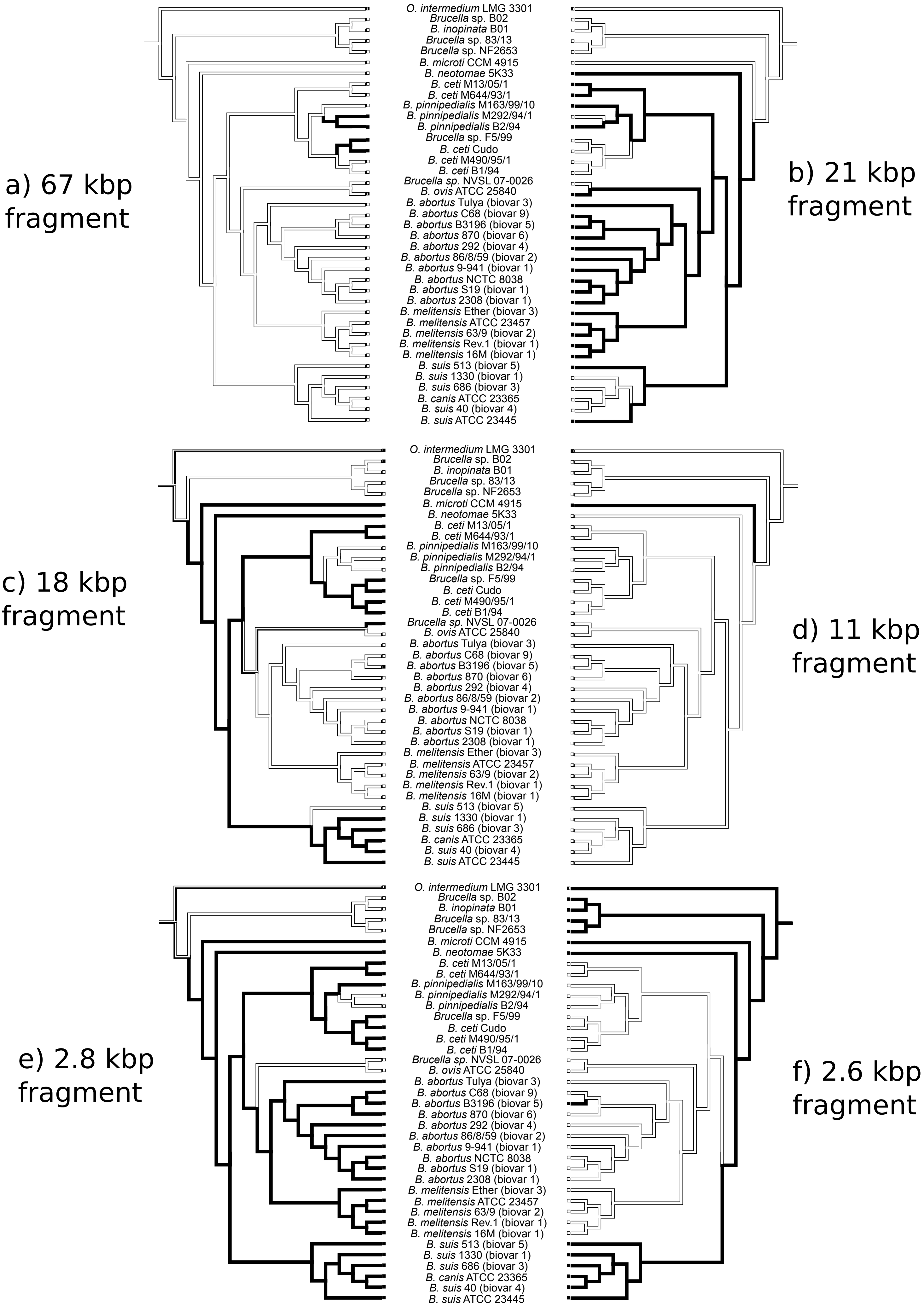
a) 67 kbp fragment
b) 21 kbp fragment
O. intermedium LMG 3301
Brucella sp. B02
B. inopinata B01
Brucella sp. 83/13
Brucella sp. NF2653
B. microti CCM 4915
B. neotomae 5K33
B. ceti M13/05/1
B. ceti M644/93/1
B. pinnipedialis M163/99/10
B. pinnipedialis M292/94/1
B. pinnipedialis B2/94
Brucella sp. F5/99
B. ceti Cudo
B. ceti M490/95/1
B. ceti B1/94
Brucella sp. NVSL 07-0026
B. ovis ATCC 25840
B. abortus Tulya (biovar 3)
B. abortus C68 (biovar 9)
B. abortus B3196 (biovar 5)
B. abortus 870 (biovar 6)
B. abortus 292 (biovar 4)
B. abortus 86/8/59 (biovar 2)
B. abortus 9-941 (biovar 1)
B. abortus NCTC 8038
B. abortus S19 (biovar 1)
B. abortus 2308 (biovar 1)
B. melitensis Ether (biovar 3)
B. melitensis ATCC 23457
B. melitensis 63/9 (biovar 2)
B. melitensis Rev.1 (biovar 1)
B. melitensis 16M (biovar 1)
B. suis 513 (biovar 5)
B. suis 1330 (biovar 1)
B. suis 686 (biovar 3)
B. canis ATCC 23365
B. suis 40 (biovar 4)
B. suis ATCC 23445
c) 18 kbp fragment
d) 11 kbp fragment
O. intermedium LMG 3301
Brucella sp. B02
B. inopinata B01
Brucella sp. 83/13
Brucella sp. NF2653
B. microti CCM 4915
B. neotomae 5K33
B. ceti M13/05/1
B. ceti M644/93/1
B. pinnipedialis M163/99/10
B. pinnipedialis M292/94/1
B. pinnipedialis B2/94
Brucella sp. F5/99
B. ceti Cudo
B. ceti M490/95/1
B. ceti B1/94
Brucella sp. NVSL 07-0026
B. ovis ATCC 25840
B. abortus Tulya (biovar 3)
B. abortus C68 (biovar 9)
B. abortus B3196 (biovar 5)
B. abortus 870 (biovar 6)
B. abortus 292 (biovar 4)
B. abortus 86/8/59 (biovar 2)
B. abortus 9-941 (biovar 1)
B. abortus NCTC 8038
B. abortus S19 (biovar 1)
B. abortus 2308 (biovar 1)
B. melitensis Ether (biovar 3)
B. melitensis ATCC 23457
B. melitensis 63/9 (biovar 2)
B. melitensis Rev.1 (biovar 1)
B. melitensis 16M (biovar 1)
B. suis 513 (biovar 5)
B. suis 1330 (biovar 1)
B. suis 686 (biovar 3)
B. canis ATCC 23365
B. suis 40 (biovar 4)
B. suis ATCC 23445
e) 2.8 kbp fragment
f) 2.6 kbp fragment
O. intermedium LMG 3301
Brucella sp. B02
B. inopinata B01
Brucella sp. 83/13
Brucella sp. NF2653
B. microti CCM 4915
B. neotomae 5K33
B. ceti M13/05/1
B. ceti M644/93/1
B. pinnipedialis M163/99/10
B. pinnipedialis M292/94/1
B. pinnipedialis B2/94
Brucella sp. F5/99
B. ceti Cudo
B. ceti M490/95/1
B. ceti B1/94
Brucella sp. NVSL 07-0026
B. ovis ATCC 25840
B. abortus Tulya (biovar 3)
B. abortus C68 (biovar 9)
B. abortus B3196 (biovar 5)
B. abortus 870 (biovar 6)
B. abortus 292 (biovar 4)
B. abortus 86/8/59 (biovar 2)
B. abortus 9-941 (biovar 1)
B. abortus NCTC 8038
B. abortus S19 (biovar 1)
B. abortus 2308 (biovar 1)
B. melitensis Ether (biovar 3)
B. melitensis ATCC 23457
B. melitensis 63/9 (biovar 2)
B. melitensis Rev.1 (biovar 1)
B. melitensis 16M (biovar 1)
B. suis 513 (biovar 5)
B. suis 1330 (biovar 1)
B. suis 686 (biovar 3)
B. canis ATCC 23365
B. suis 40 (biovar 4)
B. suis ATCC 23445
