# Supplementary material for: The genome sequence of Brucella pinnipedialis B2/94 sheds light on the evolutionary history of the genus Brucella
Source: BMC Evol Biol. 2011 Jul 11;11:200. doi: 10.1186/1471-2148-11-200 (PMC3146883; doi:10.1186/1471-2148-11-200)
Supplement: Additional file 5 — Position of IS711 insertion sequences in the genome of completely sequences Brucella, B. ceti Cudo and Brucella sp. NVSL 07-0026. Position of IS711 insertion sequences in the genome of completely sequences Brucella, B. ceti Cudo and Brucella sp. NVSL 07-0026. Each IS711 element position as well as the accession number of the genome segment to which it belongs are listed in the Table. Clusters of IS711 elements are identified by a number allowing to locate them in Figure 3. [file 1471-2148-11-200-S5.DOC]

| **ID** | ***Brucella pinnipedialis* B2/94** | ***B. ceti* Cudo** | ***B. ovis* ATCC 25840** | ***Brucella* sp. NVSL 07-0026** | ***B. microti* CCM 4915** | ***B. suis* 1330** | ***B. suis* ATCC 23445** | ***B. canis* ATCC 23365** | ***B. melitensis* 16M** | ***B. melitensis* ATCC 23457** | ***B. abortus* 2308** | ***B. abortus* 9-941** | ***B. abortus* S19** |
| --- | --- | --- | --- | --- | --- | --- | --- | --- | --- | --- | --- | --- | --- |
| (64) |  |  |  | **NZ_GG770510** 260939 .. 262168 |  |  |  |  |  |  |  |  |  |
| (32) |  | **NZ_ACJD01000001** 194677 .. 196410 | **NC_009505** 33069 .. 34802 |  | **NC_013119** 33070 .. 34805 |  |  |  |  |  |  |  |  |
| (65) |  |  |  | 394002 .. 395161 |  |  |  |  |  |  |  |  |  |
| (1) | **CP002078** 149961 .. 151697 | 310874 .. 312607 | 150028 .. 151761 | 467630 .. 468855 | 151311 .. 153046 | **NC_004310** 149734 .. 151469 | **NC_010169** 146410 .. 148145 | **NC_010103** 149717 .. 151453 | **NC_003317** 1851612 .. 1853347 | **NC_012441** 149660 .. 151396 | **NC_007618** 147454 .. 149189 | **NC_006932** 151087 .. 152822 | **NC_010742** 149473 .. 151208 |
| (2) | 264799 .. 266532 |  |  |  |  |  |  |  |  |  |  |  |  |
| (40) |  |  | 240020 .. 241756 |  |  |  |  |  |  |  |  |  |  |
| (41) |  |  | 344810 .. 346543 | 642579 .. 643800 |  |  |  |  |  |  |  |  |  |
| (42) |  |  | 360842 .. 362575 |  |  |  | 356716 .. 358451 |  |  |  |  |  |  |
| (3) | 536144 .. 537877 |  |  |  |  |  |  |  |  |  |  |  |  |
| (33) |  | 662474 .. 664207 |  |  |  |  |  |  |  |  |  |  |  |
| (4) | 545951 .. 547686 | 684472 .. 686207 | 545154 .. 546889 | 844243 .. 845429 | 525776 .. 527511 | 523651 .. 525386 | 541639 .. 543372 | 522294 .. 524031 | 1851612 .. 1853347 | 543757 .. 545492 | 541567 .. 543302 | 545272 .. 547007 | 543588 .. 545323 |
| (5) | 552296 .. 554029 | 690817 .. 692550 |  |  |  |  |  |  |  |  |  |  |  |
| (6) | 579932 .. 581669 | **NZ_ACJD01000002** 26683 .. 28418 |  |  |  |  |  |  |  |  |  |  |  |
| (7) | 597977 .. 599710 | 44719 .. 46452 |  |  |  |  |  |  |  |  |  |  |  |
| (8) | 707182 .. 708915 | 153928 .. 155661 |  |  |  |  |  |  |  |  |  |  |  |
| (66) |  |  |  | 915893 .. 917071 |  |  |  |  |  |  |  |  |  |
| (82) |  |  |  |  |  |  |  |  |  | 727329 .. 729062 |  |  |  |
| (78) |  |  |  |  |  |  | 712815 .. 714548 |  |  |  |  |  |  |
| (73) |  |  |  |  | 708345 .. 710081 |  |  |  |  |  |  |  |  |
| (34) |  | 182371 .. 184106 |  |  |  |  |  |  |  |  |  |  |  |
| (43) |  |  | 733703 .. 735436 |  |  |  |  |  |  |  |  |  |  |
| (44) |  |  | 736745 .. 738478 | 1033768 .. 1034996 |  |  |  |  |  |  |  |  |  |
| (45) |  |  | 763919 .. 765652 |  |  |  |  |  |  |  |  |  |  |
| (9) | 737117 .. 738850 | **NZ_ACJD01000003** 1996 .. 3729 |  |  |  |  |  |  |  |  |  |  |  |
| (10) | 794548 .. 796281 | 59389 .. 61122 | 791804 .. 793537 |  | 771086 .. 772821 |  | 786882 .. 788615 |  |  |  |  |  |  |
| (81) |  |  |  |  |  |  |  |  | 1209125 .. 1210859 | 796262 .. 797995 |  |  |  |
| (46) |  |  | 845401 .. 847134 |  |  |  |  |  |  |  |  |  |  |
| (11) | 887758 .. 889491 |  | 885342 .. 887075 886186 .. 887919 |  |  |  |  |  |  |  |  |  |  |
| (12) | 920235 .. 921968 | 184011 .. 185744 | 918620 .. 920353 |  | 896013 .. 897747 | 891812 .. 893546 | 911491 .. 913224 | 890182 .. 891915 | 1092191 .. 1093925 | 913196 .. 914930 | 909782 .. 911517 | 913504 .. 915237 | 911805 .. 913538 |
| (83) |  |  |  |  |  |  |  |  |  |  | 910626 .. 912359 |  |  |
| (13) | 969813 .. 971546 | 233526 .. 235259 |  | 1376627 .. 1377824 | 948323 .. 950057 | 944044 .. 945778 | 963809 .. 965542 | 942495 .. 944228 | 1042546 .. 1044279 | 962839 .. 964573 | 960167 .. 961902 | 963047 .. 964782 | 961346 .. 963081 |
| (47) |  |  | 1051871 .. 1053604 |  | 1046895 .. 1048630 |  |  |  |  |  |  |  |  |
| (48) |  |  | 1110980 .. 1112712 | 1421968 .. 1423153 |  |  |  |  |  |  |  |  |  |
| (49) |  |  | 1154959 .. 1156692 |  |  |  |  |  |  |  |  |  |  |
| (14) | 1184504 .. 1186237 | 447673 .. 449406 | 1161241 .. 1162974 |  |  |  |  |  |  |  |  |  |  |
| (15) | 1252703 .. 1254436 | 515898 .. 517632 |  |  |  |  |  |  |  | 1244698 .. 1246431 |  |  |  |
| (50) |  |  | 1174245 .. 1175978 |  |  |  |  |  |  |  |  |  |  |
| (16) | 1262619 .. 1264352 | 525847 .. 527580 | 1239688 .. 1241421 |  |  |  |  |  |  |  |  |  |  |
| (17) | 1301683 .. 1303416 | 564688 .. 566421 |  |  |  |  |  |  |  |  |  |  |  |
| (74) |  |  |  |  | 1251311 .. 1253049 |  |  |  |  |  |  |  |  |
| (51) |  |  | 1335672 .. 1337407 |  |  |  |  |  |  |  |  |  |  |
| (35) |  | 672948 .. 674409 | 1386491 .. 1388224 |  |  |  |  |  |  |  |  |  |  |
| (18) | 1460011 .. 1461744 | **NZ_ACJD01000004** 49787 .. 51520 |  |  |  |  |  |  |  |  |  |  |  |
| (36) |  | **NZ_ACJD01000005** 149893 .. 151131 |  |  |  |  |  |  |  |  |  |  |  |
| (75) |  |  |  |  | 1436403 .. 1438137 |  |  |  |  |  |  |  |  |
| (52) |  |  | 1560457 .. 1562190 |  |  |  |  |  |  |  |  |  |  |
| (77) |  |  |  |  |  | 1617676 .. 1619411 |  |  |  |  |  |  |  |
| (53) |  |  | 1719665 .. 1721403 |  |  |  |  |  |  |  |  |  |  |
| (54) |  |  | 1770719 .. 1772452 | **NZ_GG770507** 171887 .. 173118 |  |  |  |  |  |  |  |  |  |
| (76) |  |  |  |  | 1823059 .. 1824794 |  |  |  |  |  |  |  |  |
| (55) |  |  | 1934063 .. 1935796 |  |  |  |  |  |  |  |  |  |  |
| (19) | 1636852 .. 1638585 | **NZ_ACJD01000006** 17431 .. 19164 |  |  |  |  |  |  |  |  |  |  |  |
| (56) |  |  | **NC_009504** 161547 .. 163280 |  |  |  |  |  |  |  |  |  |  |
| (20) | **CP002079** 153980 .. 155713 |  |  |  |  |  |  |  |  |  |  |  |  |
| (57) |  |  | 210646 .. 212379 |  |  |  | **NC_010167** 211859 .. 213594 |  |  |  |  |  |  |
| (67) |  |  |  | **NZ_GG770511** 264606 .. 265757 265127 .. 266301 |  |  |  |  |  |  |  |  |  |
| (68) |  |  |  | 319886 .. 321040 320543 .. 321753 |  |  |  |  |  |  |  |  |  |
| (21) | 244591 .. 246324 | 543999 .. 545732 |  |  |  |  |  |  |  |  |  |  |  |
| (58) |  |  | 269190 .. 270923 |  |  |  |  |  |  |  |  |  |  |
| (69) |  |  |  | 455892 .. 457108 |  |  |  |  |  |  |  |  |  |
| (22) | 355152 .. 356885 | 672780 .. 674515 |  | 934196 .. 935356 471768 .. 472956 |  |  |  |  |  |  |  |  |  |
| (59) |  |  | 436994 .. 438727 |  |  |  |  |  |  |  |  |  |  |
| (23) | 446517 .. 448250 | 764145 .. 765878 |  |  |  |  |  |  |  |  |  |  |  |
| (24) | 498056 .. 499791 | 815608 .. 817343 |  |  |  |  |  |  |  |  |  |  |  |
| (70) |  |  |  | 755467 .. 756624 |  |  |  |  |  |  |  |  |  |
| (25) | 507328 .. 509061 |  | 494243 .. 495976 |  |  |  |  |  |  |  |  |  |  |
| (26) | 518815 .. 520548 | 835523 .. 837258 | 505729 .. 507462 | 775890 .. 777088 | **NC_013118** 532782 .. 534516 | **NC_004311** 531339 .. 533073 | 531937 .. 533670 | **NC_010104** 531324 .. 533058 | **NC_003318** 756895 .. 758629 | **NC_012442** 511410 .. 513145 | **NC_007624** 700792 .. 702527 | **NC_006933** 700804 .. 702539 | **NC_010740** 700007 .. 701742 |
| (27) | 526687 .. 528420 |  |  |  |  |  |  |  |  |  |  |  |  |
| (28) | 586204 .. 587937 | 901795 .. 903074 | 532250 .. 533983 | 775890 .. 777088 | 532782 .. 534516 | 531339 .. 533073 | 531937 .. 533670 | 531324 .. 533058 | 756895 .. 758629 | 511410 .. 513145 | 700792 .. 702527 | 700804 .. 702539 | 700007 .. 701742 |
| (60) |  |  | 533838 .. 535571 |  |  |  |  |  |  |  |  |  |  |
| (37) |  | **NZ_ACJD01000007** 74430 .. 76163 |  |  |  |  |  |  |  |  |  |  |  |
| (79) |  |  |  |  |  |  | 679177 .. 680910 |  |  |  |  |  |  |
| (29) | 690913 .. 692646 |  |  |  |  |  |  |  |  |  |  |  |  |
| (61) |  |  | 752340 .. 754073 |  |  |  |  |  |  |  |  |  |  |
| (30) | 808379 .. 810112 |  |  |  |  |  |  |  |  |  |  |  |  |
| (31) | 888765 .. 890499 | 301199 .. 302932 | 833842 .. 835574 | 934892 .. 936080 | 833211 .. 834944 | 832098 .. 833833 | 819902 .. 821637 | 831402 .. 833136 | 464532 .. 466268 | 811299 .. 813032 | 383357 .. 385090 | 383287 .. 385020 | 382574 .. 384307 |
| (62) |  |  | 969860 .. 971593 |  |  |  | 956163 .. 957896 |  |  |  |  |  |  |
| (63) |  |  | 972452 .. 974185 |  |  |  |  |  |  |  |  |  |  |
| (38) |  | 540013 .. 541746 |  |  |  |  |  |  |  |  |  |  |  |
| (39) |  | 578618 .. 580351 577617 .. 578874 |  |  |  |  |  |  |  |  |  |  |  |
| (71) |  |  |  | 1009833 .. 1011011 |  |  |  |  |  |  |  |  |  |
| (72) |  |  |  | 1091540 .. 1092747 |  |  |  |  |  |  |  |  |  |
| (80) |  |  |  |  |  |  | 1185060 .. 1186793 |  |  |  |  |  |  |
