# Supplementary material for: The genome sequence of Brucella pinnipedialis B2/94 sheds light on the evolutionary history of the genus Brucella
Source: BMC Evol Biol. 2011 Jul 11;11:200. doi: 10.1186/1471-2148-11-200 (PMC3146883; doi:10.1186/1471-2148-11-200)
Supplement: Additional file 6 — List of genes showing a large change between B. pinnipedialis B2/94 and B. microti CCM 4915. List of genes showing a large change between B. pinnipedialis B2/94 and B. microti CCM 4915. ABC transporter genes are indicated. [file 1471-2148-11-200-S6.DOC]

| **Id *B. pinnipedialis*** | **Status in *B. pinnipedialis*** | **Id *B. microti*** | **Status in *B. microti*** | **Comment** | **Category** |
| --- | --- | --- | --- | --- | --- |
| **Chromosome 1** | | | | |  |
| BPI_I34 | + | BMI_I36 | Frameshift | Hypothetical conserved protein |  |
| BPI_I49 | Frameshift | BMI_I51 | + | Bacterial surface antigen (D15) |  |
| BPI_I73 | large change | BMI_I75 | ? | Outer membrane protein |  |
| BPI_I102 | + | BMI_I104 | STOP | Hypothetical protein |  |
| BPI_I103 | Frameshift | BMI_I105 | + | Response regulator, receiver domain containing protein |  |
| BPI_I112 | Frameshift | BMI_I114 | + | Cyclic beta 1-2 glucan synthetase |  |
| BPI_I114 | + | BMI_I116 | STOP | phenazine biosynthesis protein PhzF |  |
| BPI_I125 | Frameshift | BMI_I127 | + | TP-dependent RNA helicase, DEAD/DEAH box family |  |
| BPI_I136 | Frameshift | BMI_I138 | + | transposase orfB of IS711 insertion sequence |  |
| BPI_I147 | Frameshift | BMI_I149 | + | Malate dehydrogenase (oxaloacetate-decarboxylating) (NADP+), phosphate acetyltransferase |  |
| BPI_I151 | deletion | BMI_I153 | + | RNA methyltransferase, TrmH family |  |
| BPI_I152 | deletion | BMI_I154 | + | SAM dependent methyltransferase, putative |  |
| BPI_I183 | STOP | BMI_I185 | + | phosphoadenosine phosphosulfate reductase CysH |  |
| BPI_I193 | + | BMI_I195 | ? | hypothetical protein |  |
| BPI_I195 | Frameshift | BMI_I197 | + | bifunctional sulfate adenylyltransferase subunit 1/adenylylsulfate kinase protein CysNC |  |
| BPI_I199 | + | BMI_I201 | STOP | transcritional regulator, LuxR family |  |
| from BPI_I248 to BPI_I277 | + | NA | region absent from B. microti |  |  |
| BPI_I295 | Frameshift | BMI_I266 | + | hypothetical protein |  |
| BPI_I345 | + | BMI_I316 | Frameshift | MATE efflux family protein |  |
| BPI_I351 | + | BMI_I322 | STOP | sensor histidine kinase/response regulator |  |
| BPI_I375 | + | BMI_I346 | Frameshift | sensor protein DegS |  |
| BPI_I482 | + | BMI_I455 | Frameshift | hypothetical protein |  |
| BPI_I498 | Frameshift | BMI_I471 | + | protoheme IX farnesyltransferase CtaB |  |
| BPI_I530 | + | BMI_I503 | Frameshift | polysaccharide deacetylase |  |
| BPI_I533 | + | BMI_I506 | Frameshift | metallo-beta-lactamase family protein |  |
| BPI_I568 | interrupted by IS711 family transposase | BMI_I536 | + | phosphomannomutase ManB |  |
| BPI_I575 | Frameshift | BMI_I541 | + | ribose ABC transporter, ATP-binding protein | ABC |
| BPI_I588 | + | BMI_I554 | Frameshift | L-asparaginase II protein |  |
| BPI_I592 | Frameshift | BMI_I558 | + | transporter, CorA family |  |
| BPI_I633 | Frameshift | BMI_I595 | + | hypothetical protein |  |
| BPI_I669 | Frameshift | BMI_I632 | + | hypothetical protein |  |
| BPI_I690 | Frameshift | BMI_I654 | + | coproporphyrinogen III oxidase HemN |  |
| BPI_I767 | STOP | BMI_I728 | + | transposition protein |  |
| BPI_I770 | + | BMI_I731 | Frameshift | hypothetical protein |  |
| BPI_I780 | Frameshift | BMI_I741 | + | amino acid ABC transporter, permease protein | ABC |
| BPI_I833 | + | BMI_I794 | STOP | hypothetical protein |  |
| BPI_I915 | + | BMI_I876 | Frameshift | beta-hexosaminidase A |  |
| BPI_I922 | large change | BMI_I883 | + | stationnary phase survival protein SurE |  |
| BPI_I959 | + | BMI_I918 | Frameshift | IS711, transposase orfB |  |
| BPI_I988 | STOP | BMI_I947 | + | membrane protein involved in aromatic hydrocarbon degradation |  |
| BPI_I990 | truncated | BMI_I949 | + | glutathione S-transferase domain protein |  |
| BPI_I991 | truncated | BMI_I953 | + | amino acid ABC transporter, periplasmic amino acid-binding protein | ABC |
| BPI_I1005 | Frameshift | BMI_I967 | + | site-specific recombinase, phage integrase family |  |
| BPI_I1010 | Frameshift | BMI_I972 | + | outer membrane protein |  |
| BPI_I1015 | pseudogene | BMI_I977 | also pseudogene | queuine tRNA-ribosyltransferase pseudogene |  |
| BPI_I1017 | + | BMI_I979 | Frameshift | IS711 transposase orfB |  |
| BPI_I1018 | + | BMI_I980 | Frameshift | hypothetical protein |  |
| BPI_I1075 | Frameshift | BMI_I1037 | + | hypothetical protein |  |
| BPI_I1095 | Frameshift | BMI_I1057 | + | ATP-dependent RNA helicase, DEAD/DEAH box family |  |
| BPI_I1098 | STOP | BMI_I1064 | + | drug resistance transporter, EmrB/QacA family |  |
| BPI_I1126 | Frameshift | BMI_I1090 | + | hypothetical protein |  |
| BPI_I1137 | + | BMI_I1102 | Frameshift | TetR family transcriptional regulator |  |
| BPI_I1174 | + | BMI_I1139 | Frameshift | branched-chain alpha-keto acid dehydrogenase subunit E2 |  |
| BPI_I1209 | Frameshift | BMI_I1174 | + | hypothetical protein |  |
| BPI_I1224 | Frameshift | BMI_I1189 | + | hypothetical protein |  |
| BPI_I1236 | + | BMI_I1199 | STOP | propionyl-CoA carboxylase beta chain PpcB |  |
| BPI_I1380 | STOP | BMI_I1339 | + | hypothetical protein |  |
| BPI_I1392 | Frameshift | BMI_I1352 | + | sugar ABC transporter, permease protein | ABC |
| BPI_I1404 | Frameshift | BMI_I1364 | + | hypothetical protein |  |
| BPI_I1419 | + | BMI_I1379 | Frameshift | CrcB family protein |  |
| BPI_I1426 | STOP | BMI_I1386 | + | Endonuclease/exonuclease/phosphatase family protein |  |
| BPI_I1431 | Frameshift | BMI_I1391 | + | transcriptional regulator, TetR family |  |
| BPI_I1443 | STOP | BMI_I1403 | + | hypothetical protein |  |
| BPI_I1462 | Frameshift | BMI_I1422 | + | oxidoreductase, FAD-binding |  |
| BPI_I1511 | STOP | BMI_I1471 | + | hypothetical protein |  |
| BPI_I1606 | Frameshift | BMI_I1566 | + | aspartyl/asparaginyl beta-hydroxylase |  |
| BPI_I1637 | + | BMI_I1597 | Frameshift | dipeptide transport system permease protein DppC |  |
| BPI_I1641 | + | BMI_I1601 | Frameshift | hypothetical protein |  |
| BPI_I1674 | Frameshift | BMI_I1635 | + | 7-alpha-hydroxysteroid dehydrogenase |  |
| BPI_I1700 | Frameshift | BMI_I1661 | + | transcriptional regulator, IclR family |  |
| BPI_I1708 | Frameshift | BMI_I1669 | + | malate synthase |  |
| BPI_I1735 | pseudogene | BMI_I1694 | also pseudogene | efflux transporter, RND family |  |
| BPI_I1767 | + | BMI_I1726 | Frameshift | OpgC protein |  |
| BPI_I1808 | Frameshift | BMI_I1767 | + | pyruvate kinase |  |
| BPI_I1818 | ? | BMI_I1778-9 | frameshift merge both reading frames | thiamin ABC transporter, membrane component and ATP-binding component |  |
| BPI_I1913 | + | BMI_I1873 | Frameshift | hypothetical protein |  |
| BPI_I1959 | STOP | BMI_I1921 | + | glyoxalase family protein |  |
| BPI_I2072 | Frameshift | BMI_I2035 | + | outer membrane autotransporter |  |
| BPI_I2093 | deletion | BMI_I2056 | + | cobalamin synthesis protein P47K |  |
| BPI_I2094 | + | BMI_I2058 | Frameshift | amidohydrolase |  |
| BPI_I2109 | Frameshift | BMI_I2073 | + | bacterial luciferase family protein |  |
| BPI_I2190 | + | BMI_I2154 | Frameshift | Mg chelatase-related protein |  |
| BPI_I2210 | + | BMI_I2174 | STOP | RNA modification protein |  |
|  |  |  |  |  |  |
| **Chromosome 2** | | | | |  |
| BPI_II22 | Frameshift changes end of gene | BMI_II22 | + | extracytoplasmic function alternative sigma factor |  |
| BPI_II24 | Frameshift | BMI_II24 | + | branched chain amino acid ABC transporter, periplasmic acid-binding protein | ABC |
| BPI_II32 | STOP | BMI_II32 | + | acetoin dehydrogenase, alpha/beta subunit |  |
| BPI_II85 | + | BMI_II85 | Deleted fragment | Hypothetical protein |  |
| BPI_II92 | Frameshift | BMI_II92 | + | molybdenum ABC transporter, ATP-binding protein | ABC |
| BPI_II95 | pseudogene | BMI_II95 | also pseudogene | pseudogene corresponding to Meso_1225 in Mesorhizobium sp. BNC1, hypothetical protein |  |
| BPI_II117 | + | BMI_II117 | Frameshift | HlyD family secretion protein |  |
| BPI_II120 | + | BMI_II120 | + | Probably mistakenly annotated as pseudogene in B. microti |  |
| BPI_II146 | Frameshift + in-frame change | BMI_II146 | + | hypothetical protein |  |
| BPI_II159 | + | BMI_II159 | STOP | hypothetical protein |  |
| BPI_II171 | + | BMI_II170 | larger | outer membrane autotransporter |  |
| BPI_II178 | Frameshift | BMI_II177 | + | glycolate oxidase, subunit GlcD |  |
| BPI_II188 | STOP | BMI_II187 | + | glucose/galactose transporter |  |
| BPI_II222 | Frameshift | BMI_II221 | + | CAIB/BAIF family protein |  |
| BPI_II237 | Frameshift | BMI_II236 | + | malate/L-lactate dehydrogenase family protein |  |
| BPI_II241 | STOP | BMI_II240 | + | cytochrome c oxidase, subunit III, NorE |  |
| BPI_II272 | Frameshift | BMI_II269 | + | nitrous oxide reductase regulatory protein NosR |  |
| BPI_II274 | Frameshift | BMI_II271 | + | copper ABC transporter, periplasmic copper-binding protein NosD | ABC |
| BPI_II281 | Frameshift | BMI_II278 | + | NnrS family protein, uncharacterized protein involved in response to NO |  |
| BPI_II326 | + | BMI_II323 | Frameshift | spermidine/putrescine ABC transporter membrane protein | ABC |
| BPI_II382 | Frameshift | BMI_II397 | + | branched-chain amino acid ABC transporter, periplasmic amino acid-binding protein | ABC |
| BPI_II385 | + | BMI_II400 | annotated as pseudogene in B. microti, but not difference | 4,5,-dihydroxyphthalate dehydrogenase |  |
| BPI_II393 | Frameshift | BMI_II408 | + | ROK family protein |  |
| BPI_II424 | Frameshift | BMI_II439 | + | uracil-xanthine permease |  |
| BPI_II447 | + | BMI_II462 | STOP | daunorubicin resistance transmembrane protein |  |
| BPI_II453 | Frameshift | BMI_II468 | + | transporter |  |
| BPI_II470 | Frameshift | BMI_II483 | + | glycosyl hydrolase, family 25 |  |
| BPI_II699 | + | BMI_II640 | Frameshift | 3-oxoadipate enol-lactone hydrolase PcaL |  |
| BPI_II701 | + | BMI_II642 | Frameshift | protocatechuate 3,4-dioxygenase beta ubunit PcaH |  |
| BPI_II704 | + | BMI_II645 | Frameshift | amino acid ABC transporter, periplasmic amino-acid binding protein | ABC |
| BPI_II706 | Frameshift | BMI_II647 | + | branched-chain amino acid ABC transporter, permease protein | ABC |
| BPI_II717 | + | BMI_II656 | deletion | TrkA family protein |  |
| BPI_II744 | + | BMI_II683 | ? | hypothetical protein |  |
| BPI_II748 | Frameshift | BMI_II687 | + | sugar ABC transporter, periplasmic sugar-binding protein | ABC |
| BPI_II756 | + | BMI_II695 | Frameshift | ABC transporter related protein | ABC |
| BPI_II768 | pseudogene | BMI_II707 | also pseudogene | myo-inositol catabolism IolC protein |  |
| BPI_II774 | + | BMI_II711 | STOP | myo-inositol-1(or 4)-monophosphatase |  |
| BPI_II800 | Frameshift | BMI_II739 | + | oxidoreductase, short chain dehydrogenase/reductase family |  |
| BPI_II849 | Frameshift | BMI_II786 | + | peptidase, S24 family |  |
| BPI_II851 | Frameshift | BMI_II788 | + | acetyl-CoA acetyltransferase |  |
| BPI_II901 | + | BMI_II838 | STOP | hypothetical protein |  |
| BPI_II904 | Frameshift | BMI_II841 | + | IS711 transposase OrfB |  |
| BPI_II916 | Frameshift | BMI_II853 | + | ribose ABC transporter, permease protein | ABC |
| BPI_II946 | + | BMI_II883 | Frameshift | Methylated DNA-protein cysteine methyltransferase |  |
| BPI_II959 | Frameshift | BMI_II896 | + | oxidoreductase, FMN-binding |  |
| BPI_II980 | Frameshift | BMI_II917 | + | alanine racemase |  |
| BPI_II985 | STOP | BMI_II922 | + | N-formimino-L-glutamate deiminase |  |
| BPI_II998 | STOP | BMI_II935 | + | D-galactose 1-dehydrogenase |  |
| BPI_II1001 | Frameshift | BMI_II938 | + | transcriptional regulator, GntR family |  |
| BPI_II1010 | Frameshift | BMI_II947 | + | branched-chain amino acid ABC transporter, periplasmic amino acid-binding protein | ABC |
| BPI_II1014 | Frameshift | BMI_II951 | + | branched-chain amino acid ABC transporter, permease protein | ABC |
| BPI_II1018 | Frameshift | BMI_II955 | + | ABC transporter, ATP-binding protein | ABC |
| BPI_II1041 | STOP | BMI_II978 | + | transcriptional regulator, MarR family |  |
| BPI_II1051 | Frameshift | BMI_II988 | + | ribose ABC transporter, ATP-binding protein | ABC |
| BPI_II1063 | pseudogene | BMI_II1000 | also pseudogene | glutamyl-tRNA(gln) amidotransferase subunit A |  |
| BPI_II1075 | Frameshift | BMI_II1012 | + | hypothetical protein |  |
| BPI_II1143 | + | BMI_II1084 | Frameshift | alpha/beta hydrolase fold:biotin/lipoyl attachment:esterase/lipase/thioesterase, active site, 2-oxo acid dehydrogenase |  |
| BPI_II1143 | truncated | BMI_II1086 | + | 2-oxoisovalerate dehydrogenase alpha and beta subunit |  |
| BPI_II1144 | truncated | BMI_II1088 | + | enoyl-CoA hydratase |  |
| BPI_II1180 | + | BMI_II1124 | Frameshift | N-acetylglucosamine kinase |  |
| BPI_II1188 | + | BMI_II1132 | Frameshift | hypothetical protein |  |
| BPI_II1200 | Frameshift | BMI_II1144 | Frameshift | flagellar hook-associated protein FlgK |  |
| BPI_II1220 | Frameshift | BMI_II1164 | + | 2-keto-4-pentenoate hydratase |  |
| BPI_II1256 | Frameshift | BMI_II1200 | + | branched-chain amino acid ABC transporter, permease protein | ABC |
| BPI_II1257 | Frameshift | BMI_II1201 | + | high-affinity branched-chain amino acid transport system permease protein | ABC |
| BPI_II1260 | Frameshift | BMI_II1204 | + | cadmium-translocating P-type ATPase |  |
